# Modeling human temperament and character on the basis of combined theoretical approaches

**DOI:** 10.1186/s12991-019-0247-1

**Published:** 2019-09-17

**Authors:** Konstantinos N. Fountoulakis, Xenia Gonda

**Affiliations:** 1 3rd Department of Psychiatry, School of Medicine, General Hospital AHEPA, Aristotle University of Thessaloniki, 1 Kyriakidi Street, 24636 Thessaloniki, Greece; 2 Department of Psychiatry and Psychotherapy, Semmelweis University, Kútvölgyi út 4, Budapest, 1125 Hungary; 3 MTA-SE Neuropsychopharmacology and Neurochemistry Research Group of the Hungarian Academy of Sciences and Semmelweis University, Budapest, Hungary; 4 NAP-2-SE New Antidepressant Target Research Group, Hungarian Brain Research Program, Semmelweis University, Budapest, Hungary

**Keywords:** Temperament, Personality, Model, Affective disorders, Combined theoretical approach

## Abstract

**Background:**

Although there are several models on the structure of human temperament, character and personality, the majority follow a single approach, providing a unilateral and overly theoretical construct which is unsuitable for clinical application. The current study aimed to develop a complex and comprehensive model of temperament and character by empirically combining relevant existing theories.

**Methods:**

The study included 734 healthy general population subjects aged 40.80 ± 11.48 years, who completed the TEMPS-A, TCI and NEO-PI-3 questionnaires. Data were analyzed in a multistep approach using Exploratory Factor analysis and forward stepwise linear regression.

**Results:**

The results yielded two highest order factors (Self and Self–Environment Interaction), six middle order factors (Emotional Self, Cognitive Self, Social Emotionality, Emotional and Cognitive Control, Ethical Emotionality and Behavior, Social Emotionality and Behavior) and 12 factors at the bottom (Ego Resiliency, Ego Strength, Intrapersonal Emotion, Personal Space Cognition, Interpersonal Cognition, Emotional Creativity, Externalized Interpersonal Emotion, Internalized Interpersonal Emotion, Emotional Motivation, Self-Discipline, Ethical Values and Ethical Behavior).

**Conclusions:**

The current study developed a complex hierarchical model of temperament and character on the basis of empirical data from several temperament theories. An important feature of the new temperamental model is the frequent admixture of emotional and cognitive processes within the same module. This model expands the field to include elements probably corresponding to meta-cognition mechanisms and complex interactions between affective and cognitive control, which may provide useful in understanding and treating affective disorders as well.

## Introduction

The concept of temperament is one of the most ancient in the history of the study of human behavior. It originally referred to those aspects of personality that are innate, rather than learned, whereas character was considered to be what we made of ourselves intentionally. Later it was considered that temperament is the emotional core of personality that is moderately stable throughout life, whereas character reflects a person’s goals and values as they develop over the lifespan [1]. The oldest theoretical approach included an early biological model which suggested that bodily humors were responsible for human mental health, disease and behavior. It seems that early versions of this theory might had existed in ancient Egypt or Mesopotamia, but the theory was developed in full by the school of Cos and specifically by Polybos, a pupil and son-in-law to Hippocrates (fourth century B.C.)

Temperament and personality theories influenced philosophical thinking and played a predominant role in the shaping of the anthropological and humanitarian sciences. Prominent scholars like Ernst Platner (1744–1818), Immanuel Kant (1724–1804), Friedrich Schiller (1759–1805), Friedrich Wilhelm Nietzsche (1844–1900), Rudolf Steiner (1861–1925), Ernst Kretschmer (1888–1964), and Erich Fromm (1900–1980), Emil Kraepelin (1909–1915), Alfred Adler (1879–1937), Eduard Spränger (1882–1963), William James (1842–1910), and Ernst Kretschmer (1888–1964) all developed theories of human internal psychological functioning and behavior but they were not based on strict evidence. Hans Eysenck [2–11], Jeffrey Gray [12], Jerome Kagan [13–21] Robert Cloninger [22–24] and Hagop Akiskal [25–31] all developed empirical theories of temperament and character traits and dimensions. Hans Eysenck (1916–1997) was the first to analyze personality differences using an empirical/statistical method. He proposed that the basic factors were Neuroticism (tendency to experience negative emotions), Extraversion (tendency to enjoy positive events) and Psychotisism (cognitive style). Eysenck’s theory and all the theories that derived from it, concern approach/reward, inhibition/punishment, and aggression/flight [2–11]. Currently the theories of temperament and character are represented by three major questionnaires, the NEO-PI-3, the TCI and the TEMPS-A. However these are not the only ones which exist [32].

The NEO Personality Inventory (NEO-PI) was developed by Costa and McCrae. Its original version which was published in 1978, was the Neuroticism-Extroversion-Openness Inventory (NEO-I). That version was measuring only three of the Big Five personality traits [33]. It was revised in 1985 to include all five traits and subsequently was renamed the NEO Personality Inventory (NEO-PI) and it was further refined to NEO-PI-R [34]. The latest version is the NEO-PI-3, which was published in 2005 [35]. The NEO-PI-3 includes 240-items corresponding to the Big Five personality traits (Extraversion (E), Agreeableness (A), Conscientiousness (C), Neuroticism (N), and Openness to Experience (O)) and subordinate dimensions (facets). It is suitable for use with adolescents and adults (12 years or older). All items are answered on a five-point scale, ranging from “strongly disagree” to “strongly agree”.

Robert Cloninger’s psychobiological model of temperament and character is a dimensional approach to personality assessment. It led to the development of the Temperament and Character Inventory (TCI) which measures both normal and abnormal personality traits in the two major components of personality, temperament and character [22–24, 36]. It proposes the existence of four dimensions of temperament and three dimensions of character. Each reflects normally distributed quantitative traits, present in varying degrees in everyone. Temperament dimensions (novelty seeking (NS), harm avoidance (HA), reward dependence (RD), and persistence (PS), stand for styles that are moderately stable throughout life and concern automatic basic emotional responses such as anger, fear, and disgust. Character dimensions (self-directedness (SD), cooperativeness (CO), and self-transcendence (ST)), stand for individual differences in goals, values, and self-conscious emotions like shame, guilt, and empathy, and mature in a stepwise fashion.

Hagop Akiskal’s theory focused on the affective components of temperament and their relationship to mood disorders and creativity [25–28]. This theory resulted in an operationalized definition of the five affective temperaments (depressive, hyperthymic, irritable, cyclothymic and anxious) proposed as the proximal behavioral phenotypes in the pre-morbid course of affective disorders [29–31]. The original criteria of the five temperaments derived from theoretical considerations and clinical observation [31]. Later the Temperament Evaluation of Memphis, Pisa, Paris and San Diego (TEMPS) was developed as a semi-structured TEMPS-I, administered in interview format [37, 38] and as a self-rating auto-questionnaire, the TEMPS-A [39] with 109 (for men) or 110 (for women) items.

The TEMPS is different from the Temperament and Character Inventory (TCI) [40] and the Five Factor Model (NEO-PI-R) [41] in that it frames questions in the language of affectivity, it is rooted in an evolutionary biologic perspective [26] and its clinical validity has been recently supported on a genetic basis [42].

The aim of the current study was to investigate the hierarchical latent structure of temperament and character as they are assessed with the combined use of the TEMPS-A, the NEO-PI-3 and the TCI and to test whether the hierarchical model which will derive is similar or different to previously described such models.

## Materials and methods

### Material

Volunteers gathered from around the country gathered data from their region in the frame of standardizing the three instruments. The study included subjects from the general population who satisfied the following inclusion criteria:

- Age 18–70 years.
- Lack of any physical disorder (according to self-report).
- Lack of any psychotic disorder (according to self-report and clinical impression of the examiner, after using a short interview).

The study sample included 734 subjects from the general Greek population (436 females; 59.4% and 298 males; 40.6%). Mean age was 40.80 ± 11.48 years (range 25–67 years). The mean age for females was 39.43 ± 10.87 years (range 25–65 years) while the mean age for males was 42.82 ± 12.06 years (range 25–67 years).

All subjects provided written informed consent and the protocol was approved by the Ethics committee of the Faculty of Medicine, Aristotle University of Thessaloniki, Greece.

### Methods

The protocol included the gathering of sociodemographic data and the application of the TEMPS-A [25, 43–45], the TCI [36, 46–49] and the NEO-PI-3 R [34, 35]. On the basis of this dataset all of them were officially validated in the Greek language [50–52] and their psychometric properties can be found in the related publications. It is also important to note that the collection of the data has been completed by 2008, before the current economic crisis began, and originally was used for the validation of these instruments.

### Statistical analysis

The statistical analysis included the following:

- The creation of descriptive statistics tables concerning age, gender and occupational status distribution in the sample.
- Exploratory Factor Analysis (EFA; with varimax normalized rotation) for the identification of latent structures of variables. The sample size is adequate since the maximum number of variables used in this analysis was 63 (sample size was > 10 times the number of variables). The eigenvalue > 1 was used as the criterion to select factors. In order to attribute a variable to a specific factor the loading was taken into consideration. In case there was a single loading above 0.5 this was considered to be the sole significant. For variables with lower loadings the pattern of loading was taken into consideration. This was done in two separate EFAs:
  - The first EFA analysis included all TEMPS temperament subscales and all NEO-PI-3 domains (N, E, O, A and C) and TCI temperament and character traits (high order traits; HA, NS, RD, PS, SD, CO and ST).
  - The second EFA analysis included all TEMPS temperament subscales and all NEO-PI-3 facet scales (N1-6, E1-6, O1-6, A1-6 and C1-6) and TCI temperament and character facets (lower order traits; HA1-4, NS1-4, RD1-4, PS, SD1-5, CO1-5 and ST1-3).

Both EFA analyses led to the recognition of first-order factors. Following this, the factor scores were calculated for each subject by multiplying factor loadings with values of each variable and summing the results. The factor scores underwent EFA again to investigate for the presence of second-order factors. The same procedure was performed for a third time to identify third-order factors. The above analysis was performed twice, separately one time for each EFA analysis mentioned above.

- Forward Stepwise Linear Regression Analysis (FSLRA) to test for the ability to calculate the score of one subscale on the basis of the scores of subscales from the other instruments. The components used in this analysis were all TEMPS temperament subscales and all NEO-PI-3 domains (N, E, O, A and C) and TCI temperament and character traits (high order traits; HA, NS, RD, PS, SD, CO and ST).
- Pearson Correlation coefficients were also calculated to investigate the relationship among subscales. The same components as in FSLRA were used to calculate correlations.

Data contained in this study are available from the authors on request.

## Results

### Demographic results and description of the study sample

Age and gender distribution in the study sample, compared to the general Greek population according to the 2009 census is shown in Additional file 1: Table S1. The distribution of occupation in the study sample (data were available for 533 subjects) is shown in Additional file 1: Table S2. The results indicate that the study sample is representative of the country’s active population with some over-representation of younger ages in terms of the country’s population of the year 2008, when the data were collected.

### Exploratory factor analysis results

The first EFA analysis returned four first-order factors and explained 68% of observed variance. The first factor included TEMPS depressive, cyclothymic, irritable and anxious temperaments, NEO-PI-3 N, and TCI HA and SD (the last with an opposite sign to the rest). The second factor included NEO-PI-3 E and O and TCI NS and RD. The third included TEMPS-hyperthymic temperament, NEO-PI-3 C and TCI ST. The fourth included TEMPS irritability, and with an opposite sign NEO-PI-3 A and TCI CO (Additional file 1: Table S3). The EFA with factor scores revealed the presence of two second-order factors; the first includes factor 1 and 3 and the second includes 2 and 4. It explained 50% of observed variance (Additional file 1: Table S4).

The second EFA analysis returned twelve factors and explained 63% of total variance, which is similar to the first EFA. The results are shown in Additional file 1: Table S5. The first factor included all TEMPS temperaments, NEO-PI-3 N1-4, N6, E6, and TCI HA1-4, SD1-5. The second included TEMPS-hyperthymic temperament, NEO-PI-3 C1-5, E3-4 and TCI Perseveration. The third included NEO-PI-3 A4, N5, C6, and TCI NS2-4, and SD5, the fourth included NEO-PI-3 E1, A1-6, C3, and TCI CO4, the fifth TEMPS-hyperthymic temperament, NEO-PI-3 O6 and TCI ST1-3, the sixth included NEO-PI-3 O1-3, O5-6 and E5, the seventh included TCI RD3, SD5 and CO1-3, the eighth included NEO-PI-3 E2, E6, RD2, the ninth included NEO-PI-3 O4 and TCI NS1, RD1, the tenth included TCI RD3, SD2, the eleventh was a residual factor (with highest loadings for NEO-PI-3-A1, Ε3 and Ν4) with very low loadings while the twelfth included TCI C5. The EFA with factor scores revealed the presence of five second-order factors and explained 40% of variance (Additional file 1: Table S6). An additional EFA on factor scores returned two third-order factors and explained 40% of observed variance (Additional file 1: Table S7).

**Fig. 1:**
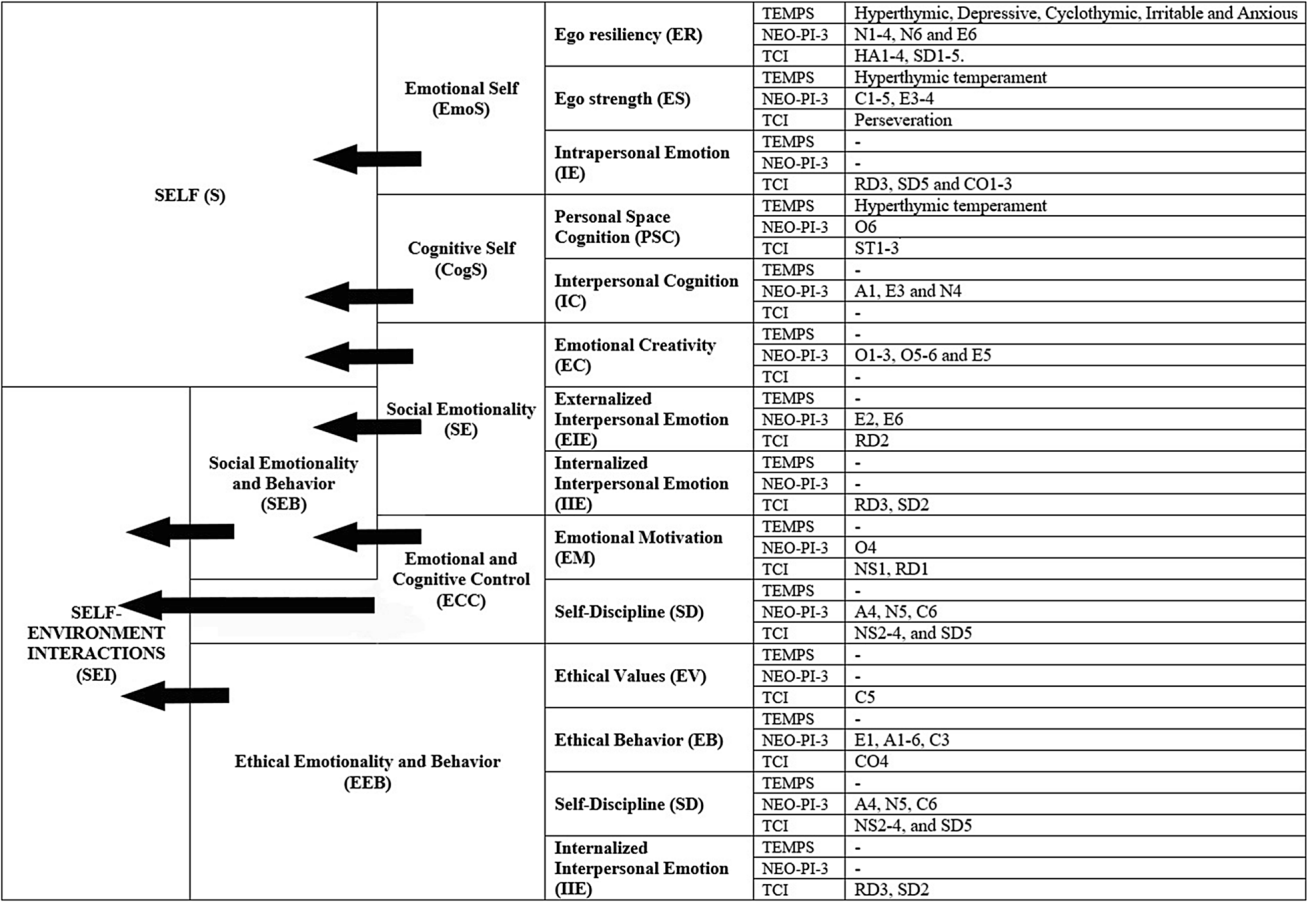
Two-dimensional visual representation of the hierarchical model of temperament

**Fig. 2:**
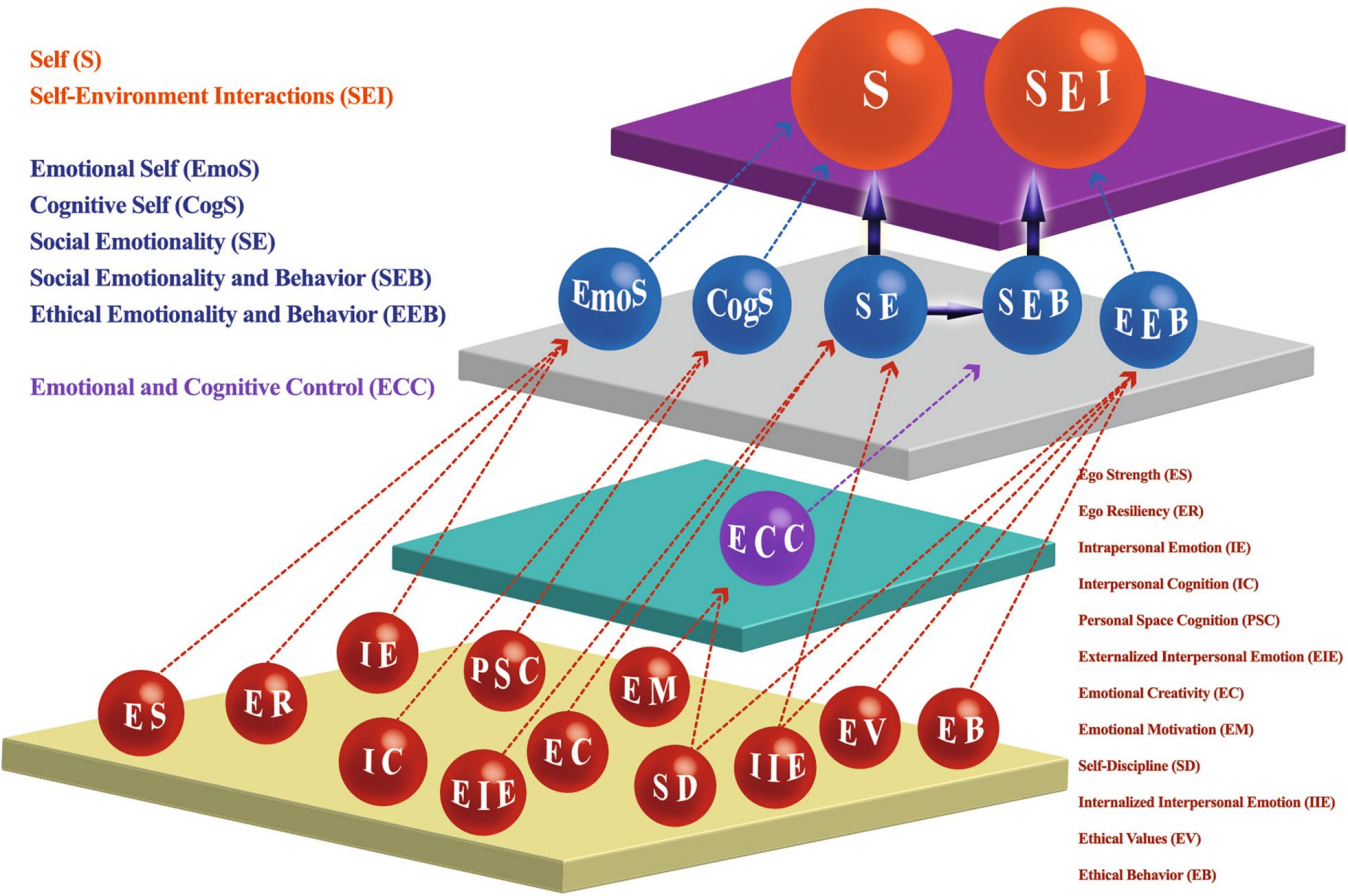
Three-dimensional visual representation of the hierarchical model of temperament

Results of the EFA analyses and the hierarchical model which emerges are shown in Figs. 1 and 2.

The correlation matrix is shown in Additional file 1: Table S8.

### Forward stepwise linear regression analysis results

The FSLRA results are shown in Additional file 1: Table S9. Highest explained percentage of variability (R^2^) was 52% while the lowest was 7%. Individual TEMPS subscales were predicted by NEO-PI-3 and TCI subscales, with explained variability 33–44% and 37–49%, respectively. The NEO-PI-3 subscales were predicted by TEMPS and TCI subscales with explained variability 4–52% and 22–54%, respectively. The TCI subscales were predicted by TEMPS and NEO-PI-3 subscales with explained variability 10–54% and 7–51%, respectively.

## Discussion

The results of the current study suggest that all TEMPS temperament subscales and all NEO-PI-3 domains (N, E, O, A and C) and TCI temperament and character traits (high order traits; HA, NS, RD, PS, SD, CO and ST) can be grouped into four factors explaining 68% of observed variance (first EFA analysis). A second EFA analysis showed that the TEMPS temperament subscales and all NEO-PI-3 facet scales (N1-6, E1-6, O1-6, A1-6 and C1-6) and TCI temperament and character facets (lower order traits; HA1-4, NS1-4, RD1-4, PS, SD1-5, CO1-5 and ST1-3) are grouped in twelve factors explaining 63% of total variance. These latter factors can be further grouped into five second-order factors explaining 40% of variance. Therefore, as shown in Figs. 1 and 2, the above analyses can provide data to support the hierarchical positioning of all the above subscales into two levels. The four factors of the first EFA analysis largely identify with the five second-order factors of the second EFA analysis. The next step of the analysis returned two second-order factors for the first EFA analysis which explained 50% of observed variance. Similarly, concerning the factor scores of the second EFA analysis, further factor analysis returned also two third-order factors and explained 40% of observed variance. The second-order factors of the first EFA analysis were identical with the third-order factors of the second EFA, so it was easy to place them in the hierarchical diagram in Figs. 1 and 2.

Notably, the low ability to predict the scores of one questionnaire on the basis of the scores of the others suggests that the three questionnaires assess different and complementary aspects of temperament. This is not in contrast with the finding that there seems to be little difference among different personality questionnaires concerning their predictive validity of real-life psychological constructs [32] since these real-life constructs are too broad and of unknown reliability.

### Interpretation of the findings and development of the model

The development of the model was based on the second EFA since it is more detailed and also essentially includes conclusions from the first EFA.

#### A. The 12 basic temperament modules

##### A1 Ego Resiliency (ER)

Starting from the bottom, the first factor includes all TEMPS temperaments; the NEO-PI-3 facets which correspond to anxiety, angry hostility, cognitive aspects of depression (guilt, hopelessness and loneliness), self-consciousness (shame and embarrassment, sensitive to ridicule and prone to feelings of inferiority), vulnerability to stress and the presence of positive emotions (being cheerful and optimistic); the TCI facets corresponding to worry and pessimism with difficulty getting over humiliating and embarrassing experiences, rumination, fear of uncertainty (rarely takes risks, difficulty adapting to changes in routine), shyness with strangers, fatigability vs vigor, responsibility vs blaming, purposefulness vs lack of goal direction (can delay gratification to achieve goals vs reactiveness and empty lives), resourcefulness vs inertia (being productive, proactive, competent and innovative), self-acceptance vs self-striving (being able to accept both strengths and limitations, good self-esteem vs unrealistic fantasies) and congruent second nature vs bad habits (organized life vs. self-defeating traits and weak will).

It could be interpreted as corresponding to ‘Ego Resiliency’ that is to the ability to cope with stress and to stay healthy and functional under pressure and under demanding conditions.

##### A2 Ego Strength (ES)

The second factor includes the Hyperthymic temperament of the TEMPS; the NEO-PI-3 facets which correspond to competence (being capable, sensible, prudent, effective), order (being neat, tidy, well organized), dutifulness (being conscientious with ethical and moral principles), achievement striving (with high aspiration levels, hard-working and possibly workaholic), self-discipline (begin tasks and completes them in spite of drawbacks), assertiveness (being dominant, forceful and socially ascendant, leadership traits), activity (with high energy, fast-paced lives); TCI facet corresponding to being industrious, hard-working, persistent and stable in spite frustration and fatigue.

This factor could be interpreted as corresponding to ‘ego strength’ since it corresponds to the ability to carry activities with vitality, competence, discipline and endurance.

##### A3 Intrapersonal Emotion (IE)

The seventh factor includes the TCI facets corresponding to attachment (intimacy vs privacy, expression of experiences and feelings, warm and lasting social attachment, sensitivity to rejection and slights), congruent second nature vs bad habits (organized life vs. self-defeating traits and weak will), social acceptance vs social intolerance, empathy vs social disinterest (being able to get ‘in other peoples’ shoes’, conscious understanding of others and respect) and helpfulness vs unhelpfulness (helpful supportive encouraging reassuring vs self-centered egoistic, selfish).

It could be interpreted as corresponding to the person’s emotional attitude towards others, it reflects an intrapersonal, deeper and core functioning, attitude and needs, and a structure which can be called ‘Intrapersonal Emotion’ (IntraE).

##### A4 Personal Space Cognition (PSC)

The fifth factor includes TEMPS-Hyperthymic temperament, the NEO-PI-3 facet corresponding to readiness to reexamine social political and religious values and TCI facets corresponding to self-forgetfulness vs self-consciousness (that is the ability to absorb within self, isolate from the surroundings and be creative vs always practical, conventional, unimaginative), transpersonal identification (the position of self to the environment and the universe) and spiritual acceptance vs. rational materialism (that is supernatural beliefs vs. objective empiricism). The presence of Hyperthymic temperament in this factor probably reflects activity level rather than emotion.

Overall this factor could be interpreted as corresponding to a cognitive attitude of the person towards the environment, memories and circumstances. Although it has to do also with other people, this is not the central element of this module. The central element seems to be the world as a whole, in the way the person perceives its own personal space. Thus a label ‘Personal Space Cognition’ (PSC) would be appropriate for this module.

##### A5 Interpersonal Cognition (IC)

The 11th factor includes NEO-PI-3 facets corresponding to trust (that is others are honest and well-intentioned vs. cynical and skeptical), assertiveness (dominant, forceful and socially ascendant, leadership tendency) and self-consciousness (shame and embarrassment, sensitive to ridicule and prone to feelings of inferiority.

It could be interpreted as corresponding to the cognitive attitude towards others and labeled as ‘Interpersonal Cognition’ (IC).

##### A6 Emotional Creativity (EC)

The sixth factor includes NEO-PI-3 facets corresponding to fantasy (creativity through daydreaming), esthetics (general interest in fine arts), feelings (emotionality, intense experience of feelings, craving for excitement), openness to new and unconventional ideas and values (readiness to reexamine social political and religious values).

It could be interpreted as corresponding to a predominantly emotional component supporting creativity and thus it could be labeled as ‘Emotional Creativity’.

##### A7 Externalized Interpersonal Emotion (EIE)

The eighth factor includes the NEO-PI-3 facets corresponding gregariousness (enjoys the company of others vs loneliness), positive emotions (being cheerful and optimistic) and the TCI facet corresponding to warmth vs cold aloofness.

Altogether this structure could be interpreted as corresponding to emotional social life with others and towards socializing and interpersonal relationships. A proper label could be ‘Interpersonal Emotion’ (InterE).

##### A8 Internalized Interpersonal Emotion (IIE)

The tenth factor includes the TCI facets attachment: intimacy vs. privacy (expression of experiences and feelings, warm and lasting social attachment, sensitivity to rejection and slights) and purposefulness vs. lack of goal direction (delay gratification to achieve goals vs. reactiveness and empty lives).

It could be interpreted as corresponding to ‘emotional social life with others-attachment’ and labeled as Internalized Interpersonal Emotion (IIE).

##### A9 Emotional Motivation (EM)

The ninth factor includes the NEO-PI-3 facet action (willingness to try new activities) and the TCI facets exploratory excitability vs. stoic rigidity (Sensation seeking) and sentimentality (easily moved by emotions, high emotional expression).

This factor could be interpreted as corresponding to an emotional motivation towards activity and could be labeled as ‘Emotional Motivation’ (EM).

##### A10 Self-Discipline (SD)

The third factor includes the NEO-PI-3 facets compliance (defers to others, tend to forgive and forget, inhibits anger), impulsiveness (control of craving and urges) and deliberation (tends to think carefully before acting) and the TCI facets impulsiveness vs reflection, extravagance vs reserve (gallant, flamboyant, unrestrained), disorderliness vs regimentation (easily express anger; prefer activities without strict rules) and congruent second nature vs bad habits (strong will, focused, reliable, goal-directed not self-defeating).

This factor could be interpreted as corresponding to impulse control and self-discipline and labeled as ‘Self-Discipline’ (SD).

##### A11 Ethical Values (EV)

The 12th factor could be interpreted as corresponding to ‘Ethical values’ and includes the TCI facet Integrated conscience vs self-serving advantage (being honest, sincere, with ethical principles).

##### Ethical Behavior (EB)

A12 The fourth factor includes the NEO-PI-3 facets warmth (interpersonal intimacy, being affectionate and friendly), trust (believing that others are honest and well-intentioned vs being cynical and skeptical), straightforwardness (being frank, sincere and ingenuous vs manipulative, flattering, deceptive), altruism (concern about the welfare of others, generosity, assists others), compliance (defers to others, tends to forgive and forget, inhibits anger), modesty (being humble and modest without low self-esteem), tender Mindedness (sympathy and concern for others vs being hardheaded and unmoved by appeals to pity), dutifulness (having sense of duty and moral obligations), and the TCI facet of compassion vs revengefulness (getting over insults and unfair treatment to be constructive in relationships).

This could be interpreted as corresponding to ‘Ethical Behavior’ (EB).

#### B. The six Higher Level Modules

##### B1 Emotional Self (EmoS)

Ego Resiliency (ER), Ego Strength (ES) and Intrapersonal Emotion (IntraE) modules group together and correspond to an emotional component of self, that is the emotional processes concerning the inner experience and the needs of oneself in order to be able to stay psychologically balanced, to cope with demands and be competent, active and productive but also to keep tuned with others in an subconscious and spontaneous, unforced and casual way. Thus it could be labeled as reflecting the emotional component of self.

##### B2 Cognitive Self (CogS)

The Personal Space Cognition (PSC) and the Interpersonal Cognition (IC) modules group together and correspond to a cognitive component of self that is the cognitive processes involved in the understanding of the environment and others.

##### B3 Social Emotionality (SE)

The Emotional Creativity (EC), the Externalized Interpersonal Emotion (EIE) and the Internalized Interpersonal Emotion (IIE) group together and correspond to mechanisms of emotional processes towards social environment and social life, that is the emotional mechanisms involved in the understanding of the social life and social environment and include among others attachment/detachment, esthetics, ideas and values, environment and others. Thus it could be labeled as ‘Social Emotionality’ (SE).

##### B4 Emotional and Cognitive Control (ECC)

Emotional Motivation (EM) and part of Self-Discipline (SD) group together and correspond to the emotional together with cognitive mechanisms involved in the filtering and manifestation of behavior that determine the interaction between the individual the environment and others.

##### B5 Ethical Emotionality and Behavior (EEB)

Ethical Behavior (EB), Ethical Values (EV) and parts of Self-Discipline (SD) and Internalized Interpersonal Emotion (IIE) group together and correspond to a complex control on activity, that is the emotional together with cognitive mechanisms involved in the filtering and manifestation of behavior that determine the interaction between the individual the environment and others, with ethical values at the center.

It can be labeled Ethical Emotionality and Behavior (EEB).

The third and fourth factors from the first EFA do not correspond exactly to the second and fourth of the second EFA. The solution to this could be that there is an important loop which bridges S and SEI through SE and ECC with the presence of an intermediate module:

##### B6 Social Emotionality and Behavior (SEB)

There is no complete correspondence among the factors identified by the first and the second EFA. The main conclusion from the inspection of the differences between them is that the second factor of the first EFA includes parts of the ‘Social Emotionality’ (SE) and f Emotional and Cognitive Control (ECC). For this reason it was considered appropriate to include in the model this factor as a module reflecting ‘Social Emotionality and Behavior’ (SEB), since it includes elements of Openness, Novelty Seeking, Reward Dependence and Extraversion.

#### C. The two super-modules at the top

##### C1 Self (S)

The two groups ‘Emotional Self’ (EmoS) and ‘Cognitive Self’ (CogS) converge to create a top supergroup reflecting aspects of the inner experience and mechanisms of ‘self’ including both emotional and cognitive aspects of ‘Self’ (S).

##### C2 Self–Environment Interactions (SEI)

‘Social Emotionality and Behavior’ (SEB) groups together with ‘Ethical Emotionality and Behavior’ (EEB) to create a top supergroup reflecting aspects of ‘Self–Environment Interactions’ (SEI).

### Theoretical and clinical implications of the model

The literature suggests that aspects of human personality extending beyond temperament usually include attitudes, beliefs, goals, and values. These elements seem to develop out of evolutionarily conserved temperament systems. Personality also includes higher-level cognitive functioning relatively unique to human beings (including language, abstract thought, meta-cognition, etc.). In terms of clinical utility, temperament is mostly studied in relationship to bipolar disorder [50–64].

The model developed with the current study suggests that the basic psychological structure in humans comprises two separate super-modules placed at the top of a hierarchical structure. One reflects the perceived components of ‘self’ and the second reflects the interaction of these components with the internal representation of the environment in interaction with its properties which have been internalized and embedded in the character of the person. Both components are ‘internal’ by definition, since they reflect subjective experiences and processing of internalized descriptions and reconstructions of the environment with emphasis on the social environment. An important element is the frequent admixture of emotional and cognitive processes in the same module. Although one of these processes seems to dominate the respective module, very often a component of the other process also exists.

It is very interesting that Social Emotionality (SE) and Social Emotionality and Behavior (SEB) seem to bridge Self (S) and Self–Environment Interaction (SEI). SE contributes to S but also to SEB which in turn contributes to SEI. This ‘bridge’ denotes that the two components of ‘Self’ are kept functionally together by social emotion and the corresponding behavior corresponding mainly to parts of ‘openness’, ‘extraversion’ and ‘reward dependence’ but also with parts of ‘novelty seeking’ and ‘ (Fig. 2). In this sense cognitive processes within the S super module probably correspond to inherent pre-existing cognitive templates (biases) while the cognitive processes within the self–environment interaction) probably correspond to meta-cognition.

So far, existing models reflect processes within emotions and within cognition separately, but almost never an interaction of these two modes of psychological function. The early theories focused on activity and affective functioning which were considered to be developmentally stable. Later attention and self-control were added [65]. These later psychological functions emerged later both in evolution and also during individual development and they are probably shaped also by the environment [66, 67]. It is highly likely that the brain circuitry which serves human psychological function is extremely complex with extensive feedback, as well as with simultaneous parallel and serial processing which makes linear analysis and solutions inadequate and relatively naïve [68–70]. In this sense, the arrows used in Figs. 1 and 2 should be considering only as marking the progress from lower to higher levels of modules rather than that of direction in the flow of information which should be considered to be largely bi-directional.

There are three dominant models of temperament and personality today and there exist significant theoretical and also essential differences between these three theoretical approaches and consequently the respected questionnaires. McCrae and Costa proposed the five-factor model (Big Five) [71] which includes neuroticism, extroversion agreeableness, openness and conscientiousness and constitutes a further development of Eysenck’s theory. The older concept of ‘psychoticism’ was substituted by agreeableness and conscientiousness while openness has some degree of overlap with extroversion [72]. Their work is largely based on the classical psycholexical study by Gordon Allport and Henry Odbert [73, 74].

The work of Robert Cloninger is characterized by an attempt to intimately connect temperamental characteristics with individual differences in genetics, neurotransmitter systems, and behavioral conditioning. He described novelty seeking (anger), harm avoidance (fear), reward dependence (attachment) and persistence (ambition) [22, 36]. His research suggests that temperament components can be assessed as early as preschool age [23] and remain moderately stable throughout a person’s lifespan except for changes from behavioral conditioning [75]. A main strength of Cloninger’s Temperament and Character model [36] is that each temperament dimension was identified and characterized as a relatively ‘pure’ and independently inherited trait that can be ascribed to the basic emotions of fear, anger, attachment and ambition or determination. Fear and anger are the most basic emotions, regulating, respectively, inhibition and initiation of behavior. In the current model harm avoidance is perceived as part of ego-resiliency, that is part of the mechanisms of self to regulate feelings of danger while novelty seeking as part of mechanisms controlling activity. Reward dependence is conceptualized as a mechanism which bridges the two others, while persistence is part of ‘ego strength’.

Hagop Akiskal has conceived temperament as the affective predisposition or reactivity, based on the original descriptions by Kraepelin (1921) of fundamental states (manic or hyperthymic, irritable, cyclothymic, anxious and depressive. These are close to the classic descriptions of Kraepelin, Kretschmer and Schneider [25]. The model of Hagop Akiskal [25, 43–45] concerns exclusively the affective temperament modules and has been conceived while evaluating and observing mood patterns in clinical practice. Empirical research has confirmed the hypothesized four-dimensional factor structure of affective temperament and is in agreement with those previously proposed on clinical populations. Temperament traits according to this model also correspond to fear and anger and it is not surprising that all these temperament traits are included mainly within the ‘self’ module and more particularly within the ‘ego resiliency’ group while the Hyperthymic temperament is also part of the ‘ego-strength’. While Hyperthymic trait is exclusively within the ‘self’ module, Irritability contributes to the ‘self–environment’ module by participating in mechanisms controlling activity, but this contribution is rather weak. The place of these traits within the current model confirms that Akiskal’s model captures the basic affective style and mood pattern as well as identifies individuals with high risk for mood disorders [76–78], suicidality but also various types of psychopathology (REF: Pompili M, Rihmer Z, Akiskal H, et al. Temperaments mediate suicide risk and psychopathology among patients with bipolar disorders. Compr Psychiatry 2012;53(3): 280–5). Some studies are in accord with the ego-resiliency vs. ego strength sub-organization of affective temperaments proposed in the current model [44, 79].

While personality refers to goals, coping styles, defensive styles, motives, self-views, life stories, and identities [80], basic personality traits (e.g., extraversion or neuroticism) are essentially parts of temperament [81]. Apart from these, there are three major systems of learning and memory which play a major role in the shaping of human behavior: associative conditioning of habits and skills, declarative learning of facts, and autonoetic learning of a personal lifetime narrative (autobiography) [82–84].

The current model expands the field to include elements probably corresponding to meta-cognition mechanisms and complex interactions between affective and cognitive control on activity. It was developed exclusively after research on mentally healthy persons so it has no direct relevance to psychopathology. Future research on patient populations might provide with valuable insight concerning the areas of dysfunction in the structure of this model.

According to this model (Fig. 2), the Self (S) comprised mainly emotional (EmoS) and thought mechanisms (CogS) which seem to be relatively distinct, highly intrinsic and independent from the environment. There seems to be a significant possibility they reflect the most genetically determined traits. On the other hand, emotional functions dominate the self–environment interaction (SEI) as well as the bridging between the two super-modules, that is Self (S) and Self–Environment Interaction (SEI). The influence of ethical values (EV and EB) seems to constitute a distinct element probably influenced significantly by forces outside the person but still they are internalized. Then it is emotional function related to social tendencies (SE) which stems out of the Self and receives the influence of control mechanisms (ECC) leading to the development of a block of social emotion and behavior (SEB) which in turn is fused with ethical values (EEB) to create the SEI. Control mechanisms (ECC) seem to constitute from two distinct modules, one emotional which has to do with emotional motivation (EM) and on cognitive which probably reflects some kind of meta-cognition (SD).

The gross structure of this model suggests that at the core of psychological function are the internal emotional and cognitive processes which through social emotionality and meta-cognition determine the externalized behavior which is further shaped by internalized social factors in the form of ethical values. It is interesting that both meta-cognitive modules (ECC and EEB) are not purely cognitive but they include a strong emotional component (EM and IIE).

The presence of two super-modules at the top is in accord with previous studies, which reported similar structure but with different functions. The first such study named these super-modules as ‘alpha’ and ‘beta’ since their psychological meaning was unclear. ‘Emotional stability’ was recognized in one of them as the analog of ER, while in the other module traits of extraversion and creativity were identified probably reflecting EIE, EC and EM among others but in a very different hierarchical structure [85]. It is interesting that Digman et al. interpreting the alpha factor (which among others included emotional stability) and shares some elements with the S super-module as a ‘Social factor’ by theorizing that emotional stability and health are the direct consequence of social environment. These authors interpreted the beta factor with shares elements with the SEI as ‘personal growth and self-fulfillment related to self-actualization’ which is generally not in contrast to the findings of the current study [85]. Other authors interpreted alpha as ‘stability’ and beta as ‘plasticity’ [86] or ‘ego control’ and ‘ego resiliency’ [26, 87]. Most models suggest the presence of a module of extraversion/positive emotionality, orienting sensitivity, and affiliativeness, and of a second model reflecting negative affect versus effortful control content [88]. In general these models recognize the presence of a function of ‘effortful control’ which is similar to the EEB module of the current module while ‘Orienting Sensitivity’ could share features with SEB, but the distinction of positive vs negative affectivity modules is not in accord with our findings. The module corresponding to ‘affiliativeness’ is probably IIE and it is located at the lowest level instead of the top [89–91].

Also the literature concerning the three major theories taken together in the current paper suggests that the four-temperament model of Akiskal [26], the cube model of Cloninger [24], the five-factor model represented by the NEO-PI [92], the seven-factor model of Tellegen [93] and Cattell’s 16 factor model [94] may in fact represent different levels of an hierarchical structure of normal and pathological personality with a two-superfactor solution at the top [26, 87], a limited number of temperaments in the middle (named under many labels, but significantly overlapping) [95] and many characters [10–15] at the bottom. According to most conceptualizations, ‘Temperament’ corresponds to the ‘higher’ levels, while ‘personality’ and ‘character’ to the ‘lower’ [96]. In another approach, fear and anger could be used in a bidimensional model to describe affective temperament traits [97, 98].

An important characteristic of the current model is that it does not accept this hierarchical separation of ‘temperament’ vs. ‘character’ and locates both of them across all hierarchical levels and modules.

### Significant outcomes of the current study

1. The basic psychological structure in humans comprised two separate super-modules (self and its interaction with environmental representation).
2. The two super-modules are ‘bridged’ by social emotion.
3. Meta-cognition seems to be a significant element of temperament and this poses important conceptual questions.
4. A defining finding was the frequent admixture of emotional and cognitive processes in the same module and even in meta-cognition.
5. An important characteristic of the current model is that it does not accept the hierarchical separation of ‘temperament’ vs. ‘character’ and locates both of them across all hierarchical levels and modules.

### Strengths of the current study

1. The current study utilized a large sample of adequate size of normal individuals more or less representative of the healthy and active population of the country and it is equivalent in size and quality to the study samples of previous similar studies.
2. The use of the three major questionnaires of temperament and character make this model development unique in the literature.
3. The utilization of labeling and definitions with a slight psychodynamic orientation in comparison to previous models which were based on the previously defined nomenclature adds an additional unique feature in this model. This made possible the easier interpretation of the functioning of specific modules which had been proven more difficult in previous attempts.

### Limitations of the current study

Several limitations of the study should be mentioned. First of all, there are limitations related to the application of linear methods. The method the current model was derived (orthogonal EFA) produces modules which do not correlate to each other. Thus at each level the modules are independent. However, the connection between levels is presumed to be reciprocal although the exact power of each direction remains elusive. Thus the arrows in Figs. 1 and 2 should not be considered as representing the direction of the flow of information and effect but rather they point to the corresponding higher-level module. This reflects a weakness of linear methods and might not correspond to reality, which probably reflects non-linear dynamical systems [49].

Second, the study has limitations related to the interpretation of the structure. In the literature there is a fundamental question whether modules exist at all or it would be rather more suitable to approach psychological function as a complete indivisible pattern (trait vs. profile) [65, 99]. The question is further complicated by the fact that the modules identified in the current model (and in all models) correspond to functions not to anatomical circuits, neurotransmitters or genes. Attempts to correlate temperament isolated traits and genes were promising but so far unsuccessful probably because of complex genetic mechanisms and environmental influence [64, 99–107].

Third, the study has limitations with respect to the questionnaires used. The scales included in the current study reflect aspects of temperament, character and personality but they do not reflect all theoretical or empirical approaches. There is the possibility the model was biased towards the theories underlying these questionnaires rather than true psychological structure per se.

Future models should utilize non-linear approaches possibly with the use of network analysis and the training of neural networks. Still all these attempts will always be limited by the fact that input will be restricted to inner experience as it is perceived and described consciously by the individual.

## Supplementary information

**Additional file 1: Table S1.** Composition of the study sample in terms of gender and age in comparison to the general population according to the Greek National Statistics Service for 2009. **Table S2.** Occupation characteristics of the study sample. **Table S3.** Factor analysis of the all TEMPS temperament subscales and all NEO-PI-3 domains (N, E, O, A and C) and TCI temperament and character traits (high order traits; HA, NS, RD, PS, SD, CO and ST). **Table S4.** Factor analysis of factor scores and the results concerning second-order factors of the initial analysis with all TEMPS temperament subscales and all NEO-PI-3 domains (N, E, O, A and C) and TCI temperament and character traits (high order traits; HA, NS, RD, PS, SD, CO and ST). **Table S5.** Factor analysis with TEMPS temperament subscales and all NEO-PI-3 facet scales (N1-6, E1-6, O1-6, A1-6 and C1-6) and TCI temperament and character facets (lower order traits; HA1-4, NS1-4, RD1-4, PS, SD1-5, CO1-5 and ST1-3). **Table S6.** Second-order factors of the initial analysis with TEMPS temperament subscales and all NEO-PI-3 facet scales (N1-6, E1-6, O1-6, A1-6 and C1-6) and TCI temperament and character facets (lower order traits; HA1-4, NS1-4, RD1-4, PS, SD1-5, CO1-5 and ST1-3). **Table S7.** Third-order factors of the initial analysis with TEMPS temperament subscales and all NEO-PI-3 facet scales (N1-6, E1-6, O1-6, A1-6 and C1-6) and TCI temperament and character facets (lower order traits; HA1-4, NS1-4, RD1-4, PS, SD1-5, CO1-5 and ST1-3). **Table S8.** Pearson Correlation coefficients. Significant were those with R >0.07 at p<0.05 (in bold italics underlined). **Table S9.** Forward Stepwise Linear Regression Analysis results in order to calculate the score of one subscale on the basis of the subscales of another questionnaire. Overall the results are poor with 7-52% of variability explained.

## Data Availability

The datasets used and analyzed during the current study are available from the corresponding author on reasonable request.
