# Supplementary material for: Modeling human temperament and character on the basis of combined theoretical approaches
Source: Ann Gen Psychiatry. 2019 Sep 17;18:21. doi: 10.1186/s12991-019-0247-1 (PMC6749666; doi:10.1186/s12991-019-0247-1)
Supplement: Supplementary file 1 — Additional file 1: Table S1. Composition of the study sample in terms of gender and age in comparison to the general population according to the Greek National Statistics Service for 2009. Table S2. Occupation characteristics of the study sample. Table S3. Factor analysis of the all TEMPS temperament subscales and all NEO-PI-3 domains (N, E, O, A and C) and TCI temperament and character traits (high order traits; HA, NS, RD, PS, SD, CO and ST). Table S4. Factor analysis of factor scores and the results concerning second-order factors of the initial analysis with all TEMPS temperament subscales and all NEO-PI-3 domains (N, E, O, A and C) and TCI temperament and character traits (high order traits; HA, NS, RD, PS, SD, CO and ST). Table S5. Factor analysis with TEMPS temperament subscales and all NEO-PI-3 facet scales (N1-6, E1-6, O1-6, A1-6 and C1-6) and TCI temperament and character facets (lower order traits; HA1-4, NS1-4, RD1-4, PS, SD1-5, CO1-5 and ST1-3). Table S6. Second-order factors of the initial analysis with TEMPS temperament subscales and all NEO-PI-3 facet scales (N1-6, E1-6, O1-6, A1-6 and C1-6) and TCI temperament and character facets (lower order traits; HA1-4, NS1-4, RD1-4, PS, SD1-5, CO1-5 and ST1-3). Table S7. Third-order factors of the initial analysis with TEMPS temperament subscales and all NEO-PI-3 facet scales (N1-6, E1-6, O1-6, A1-6 and C1-6) and TCI temperament and character facets (lower order traits; HA1-4, NS1-4, RD1-4, PS, SD1-5, CO1-5 and ST1-3). Table S8. Pearson Correlation coefficients. Significant were those with R >0.07 at p<0.05 (in bold italics underlined). Table S9. Forward Stepwise Linear Regression Analysis results in order to calculate the score of one subscale on the basis of the subscales of another questionnaire. Overall the results are poor with 7-52% of variability explained. [file 12991_2019_247_MOESM1_ESM.docx]

**Additional files**

**Additional Table S1:** Composition of the study sample in terms of gender and age in comparison to the general population according to the Greek National Statistics Service for 2009

| Age group | Greek population  (approximation for 2009) | Study sample |
| --- | --- | --- |
| Total population | 11,282,751 | 734 |
| Females vs. males | 48% vs. 52% | 40.6% vs 59.4% |
| 25-29 years old | 11.02 % | 25.81 % |
| 29-34 years old | 11.31 % | 12.90 % |
| 34-39 years old | 10.00 % | 15.44 % |
| 40-44 years old | 10.00 % | 13.13 % |
| 44-49 years old | 9.21 % | 10.60 % |
| 50-54 years old | 8.92 % | 10.14 % |
| 55-59 years old | 6.83 % | 8.29 % |
| 60-64 years old | 7.09 % | 3.69 % |

**Additional Table S2:** Occupation characteristics of the study sample

|  | Count | % |
| --- | --- | --- |
| He/she used to work but is currently unemployed | 0 | 0.00 |
| He/she never worked and neither does now | 0 | 0.00 |
| Clerk (civil or private) | 338 | 63.41 |
| Free professional (tradesman, craftsman) | 62 | 11.63 |
| Doctor, lawyer, engineer, priest, teacher etc. | 75 | 14.07 |
| Student (college or university) | 12 | 2.25 |
| Blue collar worker (construction worker, farmer) | 26 | 4.88 |
| Housewife | 20 | 3.75 |
| Total | 533 | 100.00 |

**Additional Table S3:** Factor analysis of the all TEMPS temperament subscales and all NEO-PI-3 domains (N, E, O, A and C) and TCI temperament and character traits (high order traits; HA, NS, RD, PS, SD, CO and ST)

|  | 1st order factors | | | |
| --- | --- | --- | --- | --- |
|  | 1 | 2 | 3 | 4 |
| TEMPS-Depr | -0.79 | -0.28 | 0.09 | 0.15 |
| TEMPS-Cycl | -0.76 | 0.09 | 0.15 | -0.29 |
| TEMPS-Hyper | 0.34 | 0.35 | 0.63 | -0.24 |
| TEMPS-Irrit | -0.58 | 0.04 | 0.13 | -0.58 |
| TEMPS-Anx | -0.84 | -0.14 | 0.10 | -0.09 |
| NEO-PI-3-N | -0.80 | -0.03 | -0.20 | -0.19 |
| NEO-PI-3-E | 0.37 | 0.58 | 0.42 | 0.10 |
| NEO-PI-3-O | 0.00 | 0.66 | 0.11 | 0.08 |
| NEO-PI-3-A | 0.06 | -0.04 | 0.09 | 0.85 |
| NEO-PI-3-C | 0.32 | -0.25 | 0.60 | 0.40 |
| TCI-NS | 0.02 | 0.82 | -0.18 | -0.29 |
| TCI-HA | -0.73 | -0.29 | -0.34 | 0.09 |
| TCI-RD | 0.19 | 0.75 | -0.06 | 0.27 |
| TCI-PS | -0.09 | -0.14 | 0.73 | 0.13 |
| TCI-SD | 0.75 | -0.02 | 0.16 | 0.31 |
| TCI-CO | 0.16 | 0.24 | 0.09 | 0.79 |
| TCI-ST | -0.34 | 0.30 | 0.51 | -0.04 |
| Explained Variance | 4.52 | 2.54 | 2.03 | 2.33 |
| Proportion of total variance | 27% | 15% | 12% | 14% |
| Total Varance explained |  |  |  | 68% |

**Additional Table S4:** Factor analysis of factor scores and the results concerning 2nd order factors of the initial analysis with all TEMPS temperament subscales and all NEO-PI-3 domains (N, E, O, A and C) and TCI temperament and character traits (high order traits; HA, NS, RD, PS, SD, CO and ST)

|  | 2nd order factors | |
| --- | --- | --- |
|  | 1 | 2 |
| Factor 1 score | 0.83 | 0.12 |
| Factor 2 score | 0.05 | 0.79 |
| Factor 3 score | 0.54 | -0.15 |
| Factor 4 score | -0.10 | 0.58 |
| Explained Variance | 1.00 | 1.00 |
| Proportion of total variance | 25% | 25% |
| Total Varance explained |  | 50% |

**Additional Table S5:** Factor analysis with TEMPS temperament subscales and all NEO-PI-3 facet scales (N1-6, E1-6, O1-6, A1-6 and C1-6) and TCI temperament and character facets (lower order traits; HA1-4, NS1-4, RD1-4, PS, SD1-5, CO1-5 and ST1-3)

|  | 1st order factors | | | | | | | | | | | |
| --- | --- | --- | --- | --- | --- | --- | --- | --- | --- | --- | --- | --- |
|  | 1 | 2 | 3 | 4 | 5 | 6 | 7 | 8 | 9 | 10 | 11 | 12 |
| TEMPS-Depr | -0,71 | -0,03 | 0,12 | 0,11 | 0,16 | -0,04 | 0,05 | -0,25 | -0,18 | -0,16 | 0,07 | -0,08 |
| TEMPS-Cycl | -0,63 | -0,01 | -0,31 | -0,05 | 0,26 | -0,01 | -0,03 | -0,19 | 0,00 | 0,01 | 0,19 | -0,27 |
| TEMPS-Hyper | 0,40 | 0,41 | -0,19 | -0,01 | 0,40 | 0,01 | -0,02 | 0,14 | 0,14 | 0,14 | 0,16 | -0,16 |
| TEMPS-Irrit | -0,54 | 0,10 | -0,35 | -0,35 | 0,07 | 0,05 | -0,18 | -0,14 | -0,07 | 0,08 | 0,27 | -0,14 |
| TEMPS-Anx | -0,82 | 0,02 | -0,01 | -0,06 | 0,15 | -0,06 | -0,03 | -0,07 | -0,07 | -0,03 | 0,16 | -0,13 |
| NEO-PI-3-N1 | -0,79 | 0,01 | -0,12 | -0,05 | -0,05 | 0,03 | 0,04 | 0,04 | -0,07 | 0,13 | -0,16 | 0,08 |
| NEO-PI-3-E1 | 0,18 | 0,24 | -0,03 | 0,62 | 0,11 | 0,17 | 0,14 | 0,36 | -0,03 | 0,08 | 0,18 | -0,10 |
| NEO-PI-3-O1 | 0,00 | -0,21 | -0,24 | -0,18 | 0,07 | 0,62 | 0,12 | 0,08 | 0,01 | -0,02 | -0,12 | -0,14 |
| NEO-PI-3-A1 | 0,24 | -0,11 | -0,04 | 0,45 | 0,12 | 0,00 | 0,21 | 0,26 | -0,12 | -0,08 | 0,32 | 0,23 |
| NEO-PI-3-C1 | 0,29 | 0,55 | 0,23 | 0,15 | -0,10 | 0,18 | -0,09 | 0,14 | -0,14 | 0,21 | 0,22 | -0,09 |
| NEO-PI-3-N2 | -0,57 | 0,07 | -0,35 | -0,29 | 0,01 | -0,04 | -0,22 | -0,15 | -0,04 | 0,23 | -0,03 | 0,08 |
| NEO-PI-3-E2 | 0,24 | 0,13 | 0,01 | 0,15 | -0,01 | 0,04 | 0,11 | 0,70 | 0,18 | -0,11 | 0,00 | -0,11 |
| NEO-PI-3-O2 | -0,06 | 0,01 | 0,07 | 0,11 | 0,17 | 0,73 | 0,03 | 0,04 | 0,25 | 0,01 | 0,04 | 0,16 |
| NEO-PI-3-A2 | 0,02 | 0,06 | 0,22 | 0,52 | -0,17 | -0,06 | 0,12 | 0,00 | 0,00 | -0,18 | -0,10 | 0,33 |
| NEO-PI-3-C2 | -0,05 | 0,52 | 0,42 | 0,08 | -0,03 | -0,06 | -0,04 | 0,04 | 0,07 | -0,02 | -0,21 | 0,02 |
| NEO-PI-3-N3 | -0,76 | -0,16 | -0,11 | -0,01 | 0,11 | 0,02 | -0,06 | -0,18 | -0,03 | 0,01 | -0,13 | 0,11 |
| NEO-PI-3-E3 | 0,34 | 0,46 | -0,19 | -0,31 | 0,05 | 0,07 | -0,05 | 0,21 | 0,12 | 0,06 | 0,38 | 0,05 |
| NEO-PI-3-O3 | -0,08 | 0,31 | -0,23 | 0,16 | 0,03 | 0,61 | 0,10 | 0,15 | -0,02 | 0,13 | -0,07 | 0,08 |
| NEO-PI-3-A3 | 0,00 | 0,36 | 0,12 | 0,72 | -0,04 | 0,10 | 0,11 | 0,14 | 0,00 | 0,03 | 0,01 | -0,01 |
| NEO-PI-3-C3 | 0,01 | 0,57 | 0,24 | 0,48 | -0,11 | 0,10 | 0,07 | 0,02 | -0,14 | 0,07 | 0,04 | 0,12 |
| NEO-PI-3-N4 | -0,56 | -0,09 | -0,03 | 0,13 | 0,04 | -0,05 | -0,10 | -0,14 | -0,13 | 0,00 | -0,38 | 0,10 |
| NEO-PI-3-E4 | 0,18 | 0,70 | -0,20 | 0,13 | 0,10 | -0,02 | 0,01 | 0,07 | 0,08 | -0,10 | 0,01 | -0,02 |
| NEO-PI-3-O4 | 0,23 | 0,03 | -0,05 | -0,12 | -0,05 | 0,28 | 0,09 | -0,04 | 0,60 | -0,13 | -0,12 | -0,20 |
| NEO-PI-3-A4 | 0,09 | -0,23 | 0,44 | 0,54 | 0,15 | -0,13 | 0,15 | 0,05 | -0,02 | -0,23 | -0,04 | 0,01 |
| NEO-PI-3-C4 | 0,16 | 0,74 | 0,15 | 0,14 | 0,03 | 0,09 | 0,03 | 0,03 | 0,04 | 0,17 | -0,07 | -0,02 |
| NEO-PI-3-N5 | -0,35 | 0,07 | -0,59 | -0,22 | 0,13 | 0,09 | 0,06 | 0,02 | 0,05 | 0,03 | -0,24 | -0,06 |
| NEO-PI-3-E5 | 0,17 | 0,18 | -0,35 | -0,01 | 0,16 | 0,45 | -0,05 | 0,17 | 0,16 | -0,04 | -0,15 | -0,23 |
| NEO-PI-3-O5 | 0,16 | 0,09 | 0,10 | 0,00 | 0,09 | 0,69 | 0,10 | -0,12 | 0,28 | -0,07 | 0,25 | 0,07 |
| NEO-PI-3-A5 | -0,18 | -0,03 | 0,02 | 0,50 | -0,04 | -0,24 | 0,08 | -0,24 | 0,05 | 0,09 | -0,25 | 0,19 |
| NEO-PI-3-C5 | 0,22 | 0,62 | 0,42 | 0,18 | -0,05 | -0,03 | 0,12 | 0,04 | 0,05 | 0,01 | 0,04 | 0,04 |
| NEO-PI-3-N6 | -0,60 | -0,41 | -0,17 | -0,24 | 0,07 | -0,08 | -0,05 | -0,01 | 0,01 | -0,14 | -0,20 | 0,07 |
| NEO-PI-3-E6 | 0,41 | 0,14 | -0,25 | 0,29 | 0,12 | 0,26 | 0,08 | 0,39 | 0,04 | 0,06 | -0,24 | -0,25 |
| NEO-PI-3-O6 | 0,09 | -0,01 | -0,15 | 0,04 | -0,41 | 0,45 | 0,32 | -0,13 | 0,15 | 0,05 | 0,02 | -0,21 |
| NEO-PI-3-A6 | -0,04 | 0,25 | 0,03 | 0,68 | 0,00 | 0,10 | 0,09 | 0,04 | -0,07 | 0,06 | -0,04 | 0,07 |
| NEO-PI-3-C6 | 0,08 | 0,30 | 0,72 | 0,17 | -0,09 | 0,02 | -0,07 | -0,02 | -0,06 | 0,17 | 0,12 | 0,03 |
| NS1 | 0,23 | 0,09 | -0,19 | -0,02 | 0,07 | 0,20 | 0,07 | 0,24 | 0,81 | 0,11 | 0,06 | 0,01 |
| NS2 | 0,09 | -0,17 | -0,70 | 0,03 | 0,01 | -0,01 | -0,08 | 0,00 | 0,13 | -0,07 | 0,05 | -0,06 |
| NS3 | 0,00 | -0,09 | -0,51 | 0,03 | -0,09 | 0,25 | 0,02 | 0,23 | 0,14 | 0,11 | -0,01 | 0,13 |
| NS4 | -0,09 | -0,07 | -0,56 | -0,21 | -0,07 | 0,16 | -0,02 | 0,07 | 0,06 | -0,02 | 0,14 | -0,22 |
| HA1 | -0,74 | -0,09 | 0,07 | -0,08 | -0,12 | -0,03 | -0,09 | 0,01 | -0,01 | -0,15 | -0,01 | 0,06 |
| HA2 | -0,61 | -0,12 | 0,19 | 0,09 | -0,16 | -0,03 | 0,02 | 0,16 | -0,17 | 0,18 | -0,07 | 0,07 |
| HA3 | -0,46 | -0,23 | 0,09 | 0,11 | -0,16 | 0,06 | -0,20 | -0,17 | -0,13 | -0,31 | -0,23 | 0,11 |
| HA4 | -0,53 | -0,34 | 0,10 | -0,02 | -0,09 | 0,00 | -0,18 | 0,06 | -0,12 | -0,11 | 0,06 | -0,13 |
| RD1 | 0,23 | 0,09 | -0,19 | -0,02 | 0,07 | 0,20 | 0,07 | 0,24 | 0,81 | 0,11 | 0,06 | 0,01 |
| RD2 | 0,06 | 0,01 | -0,14 | 0,08 | 0,04 | 0,02 | 0,21 | 0,70 | 0,18 | 0,14 | 0,07 | 0,16 |
| RD3 | -0,08 | -0,03 | 0,02 | 0,04 | -0,25 | 0,01 | 0,40 | 0,22 | -0,08 | -0,52 | -0,08 | 0,25 |
| PER | -0,11 | 0,57 | 0,28 | -0,09 | 0,30 | 0,01 | 0,12 | -0,16 | 0,14 | -0,09 | 0,05 | 0,10 |
| SD1 | 0,57 | 0,01 | 0,12 | 0,02 | -0,23 | 0,01 | 0,23 | 0,11 | 0,07 | 0,37 | -0,08 | 0,16 |
| SD2 | 0,42 | 0,21 | 0,18 | 0,05 | -0,06 | 0,02 | 0,17 | 0,19 | 0,02 | 0,56 | -0,06 | 0,07 |
| SD3 | 0,56 | 0,30 | 0,10 | -0,12 | 0,03 | 0,04 | 0,15 | -0,01 | 0,17 | 0,35 | 0,05 | 0,14 |
| SD4 | 0,42 | -0,01 | 0,12 | 0,15 | -0,18 | -0,15 | 0,19 | -0,14 | -0,06 | 0,13 | 0,22 | 0,33 |
| SD5 | 0,34 | 0,27 | 0,37 | 0,07 | -0,04 | 0,08 | 0,36 | 0,07 | -0,05 | 0,19 | -0,16 | 0,02 |
| CO1 | 0,17 | -0,01 | 0,07 | 0,28 | 0,04 | 0,03 | 0,72 | -0,03 | 0,12 | 0,08 | 0,07 | -0,05 |
| CO2 | 0,10 | 0,08 | -0,01 | 0,07 | 0,02 | 0,21 | 0,64 | 0,13 | 0,13 | 0,02 | -0,01 | 0,06 |
| CO3 | 0,12 | 0,07 | -0,02 | 0,21 | 0,00 | 0,06 | 0,59 | 0,22 | -0,07 | -0,08 | -0,01 | 0,21 |
| CO4 | 0,15 | -0,04 | 0,20 | 0,46 | 0,19 | -0,11 | 0,34 | 0,04 | 0,04 | -0,04 | 0,05 | 0,31 |
| CO5 | 0,02 | 0,07 | 0,10 | 0,27 | 0,00 | 0,09 | 0,15 | 0,03 | -0,07 | -0,01 | -0,02 | 0,69 |
| ST1 | -0,19 | -0,03 | -0,16 | -0,05 | 0,64 | 0,28 | -0,07 | -0,14 | 0,08 | 0,04 | 0,05 | -0,18 |
| ST2 | 0,00 | 0,01 | 0,09 | 0,15 | 0,78 | -0,01 | 0,01 | 0,03 | 0,11 | 0,06 | 0,10 | 0,07 |
| ST3 | -0,09 | 0,10 | -0,05 | -0,08 | 0,64 | 0,16 | 0,11 | 0,08 | -0,10 | -0,06 | -0,17 | 0,00 |
| Expl.Var | 7,92 | 4,36 | 4,06 | 3,95 | 2,52 | 2,93 | 2,45 | 2,27 | 2,36 | 1,58 | 1,34 | 1,70 |
| Prp.Totl | 13% | 7% | 7% | 7% | 4% | 5% | 4% | 4% | 4% | 3% | 2% | 3% |
| Total variance explained |  |  |  |  |  |  |  |  |  |  |  | 63% |

**Additional Table S6:** Second order factors of the initial analysis with TEMPS temperament subscales and all NEO-PI-3 facet scales (N1-6, E1-6, O1-6, A1-6 and C1-6) and TCI temperament and character facets (lower order traits; HA1-4, NS1-4, RD1-4, PS, SD1-5, CO1-5 and ST1-3)

|  | 2nd order factor | | | | |
| --- | --- | --- | --- | --- | --- |
|  | 1 | 2 | 3 | 4 | 5 |
| 1st Order Factor 1 | 0,23 | 0,11 | 0,50 | 0,32 | 0,11 |
| 1st Order Factor 2 | -0,12 | 0,10 | 0,56 | 0,07 | 0,02 |
| 1st Order Factor 3 | 0,03 | 0,40 | 0,07 | -0,41 | 0,13 |
| 1st Order Factor 4 | -0,10 | 0,62 | 0,01 | 0,03 | -0,13 |
| 1st Order Factor 5 | -0,11 | -0,13 | 0,10 | 0,01 | 0,75 |
| 1st Order Factor 6 | 0,58 | -0,15 | -0,05 | 0,12 | -0,32 |
| 1st Order Factor 7 | 0,03 | -0,17 | 0,55 | -0,28 | -0,18 |
| 1st Order Factor 8 | 0,54 | -0,04 | 0,21 | -0,24 | 0,13 |
| 1st Order Factor 9 | -0,02 | 0,06 | 0,07 | 0,75 | 0,03 |
| 1st Order Factor 10 | 0,51 | 0,20 | -0,26 | 0,07 | 0,24 |
| 1st Order Factor 11 | -0,11 | 0,01 | 0,09 | 0,01 | -0,43 |
| 1st Order Factor 12 | 0,06 | 0,57 | 0,02 | 0,02 | -0,02 |
| Explained variable | 1,00 | 1,00 | 1,00 | 1,00 | 1,00 |
| Proportion of total | 8% | 8% | 8% | 8% | 8% |
| Total variance explained |  |  |  |  | 40% |

**Additional Table S7:** Third order factors of the initial analysis with TEMPS temperament subscales and all NEO-PI-3 facet scales (N1-6, E1-6, O1-6, A1-6 and C1-6) and TCI temperament and character facets (lower order traits; HA1-4, NS1-4, RD1-4, PS, SD1-5, CO1-5 and ST1-3)

|  | 3rd order factor | |
| --- | --- | --- |
|  | 1 | 2 |
| 2nd Order Factor 1 | 0,24 | -0,60 |
| 2nd Order Factor 2 | 0,54 | -0,06 |
| 2nd Order Factor 3 | 0,07 | 0,75 |
| 2nd Order Factor 4 | 0,79 | 0,12 |
| 2nd Order Factor 5 | -0,13 | -0,23 |
| Explained variable | 1,00 | 1,00 |
| Proportion of total | 20% | 20% |
| Total variance explained |  | 40% |

**Additional Table S8:** Pearson Correlation coefficients. Significant were those with R >0.07 at p<0.05 (in bold italics underlined)

|  | TEMPS-Depr | TEMPS-Cycl | TEMPS-Hyper | TEMPS-Irrit | TEMPS-Anx | NEO-PI-3-N | NEO-PI-3-E | NEO-PI-3-O | NEO-PI-3-A | NEO-PI-3-C | TCI-NS | TCI-HA | TCI-RD | TCI-PS | TCI-SD | TCI-CO | TCI-ST |
| --- | --- | --- | --- | --- | --- | --- | --- | --- | --- | --- | --- | --- | --- | --- | --- | --- | --- |
| TEMPS-Depr | 1.00 |  |  |  |  |  |  |  |  |  |  |  |  |  |  |  |  |
| TEMPS-Cycl | 0.56 | 1.00 |  |  |  |  |  |  |  |  |  |  |  |  |  |  |  |
| TEMPS-Hyper | -0.33 | -0.05 | 1.00 |  |  |  |  |  |  |  |  |  |  |  |  |  |  |
| TEMPS-Irrit | 0.36 | 0.62 | 0.00 | 1.00 |  |  |  |  |  |  |  |  |  |  |  |  |  |
| TEMPS-Anx | 0.64 | 0.62 | -0.25 | 0.56 | 1.00 |  |  |  |  |  |  |  |  |  |  |  |  |
| NEO-PI-3-N | 0.52 | 0.56 | -0.34 | 0.52 | 0.64 | 1.00 |  |  |  |  |  |  |  |  |  |  |  |
| NEO-PI-3-E | -0.39 | -0.18 | 0.55 | -0.19 | -0.32 | -0.40 | 1.00 |  |  |  |  |  |  |  |  |  |  |
| NEO-PI-3-O | -0.15 | -0.01 | 0.15 | -0.01 | -0.14 | -0.07 | 0.37 | 1.00 |  |  |  |  |  |  |  |  |  |
| NEO-PI-3-A | 0.07 | -0.23 | -0.04 | -0.47 | -0.12 | -0.24 | 0.17 | -0.05 | 1.00 |  |  |  |  |  |  |  |  |
| NEO-PI-3-C | -0.08 | -0.29 | 0.24 | -0.27 | -0.17 | -0.41 | 0.32 | 0.02 | 0.39 | 1.00 |  |  |  |  |  |  |  |
| TCI-NS | -0.28 | 0.13 | 0.23 | 0.17 | -0.11 | 0.05 | 0.33 | 0.39 | -0.24 | -0.36 | 1.00 |  |  |  |  |  |  |
| TCI-HA | 0.56 | 0.38 | -0.54 | 0.32 | 0.60 | 0.64 | -0.51 | -0.17 | -0.02 | -0.24 | -0.21 | 1.00 |  |  |  |  |  |
| TCI-RD | -0.32 | -0.19 | 0.19 | -0.20 | -0.24 | -0.20 | 0.48 | 0.35 | 0.13 | 0.01 | 0.52 | -0.27 | 1.00 |  |  |  |  |
| TCI-PS | 0.13 | 0.03 | 0.20 | 0.03 | 0.13 | -0.03 | 0.12 | 0.05 | 0.07 | 0.44 | -0.21 | -0.10 | -0.01 | 1.00 |  |  |  |
| TCI-SD | -0.50 | -0.56 | 0.23 | -0.51 | -0.57 | -0.63 | 0.30 | 0.07 | 0.25 | 0.46 | -0.10 | -0.58 | 0.22 | 0.09 | 1.00 |  |  |
| TCI-CO | -0.09 | -0.27 | 0.08 | -0.45 | -0.24 | -0.29 | 0.24 | 0.16 | 0.58 | 0.25 | -0.07 | -0.22 | 0.34 | 0.10 | 0.43 | 1.00 |  |
| TCI-ST | 0.18 | 0.29 | 0.29 | 0.15 | 0.21 | 0.14 | 0.13 | 0.18 | -0.02 | -0.05 | 0.10 | -0.05 | 0.03 | 0.23 | -0.20 | 0.04 | 1.00 |

**Additional Table S9:** Forward Stepwise Linear Regression Analysis results in order to calculate the score of one subscale on the basis of the subscales of another questionnaire. Overall the results are poor with 7-52% of variability explained.

| TEMPS-Depressive Temperament  R= 0.61 R²=0.38 Adjusted R²= 0.37 F(5.728)=88.683 p<0.0000  Std.Error of estimate: 2.6407 | | | | | | |
| --- | --- | --- | --- | --- | --- | --- |
|  | b* | Std.Err. | b | Std.Err. | t(728) | p-value |
| Intercept |  |  | -2.156 | 1.445 | -1.492 | 0.136236 |
| NEO-PI-3-N | 0.523 | 0.034 | 0.089 | 0.006 | 15.406 | 0.000000 |
| NEO-PI-3-E | -0.257 | 0.035 | -0.054 | 0.007 | -7.282 | 0.000000 |
| NEO-PI-3-O | -0.010 | 0.032 | -0.002 | 0.006 | -0.320 | 0.749282 |
| NEO-PI-3-A | 0.188 | 0.032 | 0.040 | 0.007 | 5.864 | 0.000000 |
| NEO-PI-3-C | 0.141 | 0.034 | 0.025 | 0.006 | 4.095 | 0.000047 |
|  |  |  |  |  |  |  |
| TEMPS-Cyclothymic Ttemperament  R=0.57 R²=0.33 Adjusted R²=0.33 F(5.728)=72.632 p<0.0000  Std.Error of estimate: 3.6953 | | | | | | |
| Intercept |  |  | -1.967 | 2.022 | -0.973 | 0.331009 |
| NEO-PI-3-N | 0.548 | 0.035 | 0.126 | 0.008 | 15.593 | 0.000000 |
| NEO-PI-3-E | 0.077 | 0.037 | 0.022 | 0.010 | 2.097 | 0.036348 |
| NEO-PI-3-O | -0.004 | 0.033 | -0.001 | 0.009 | -0.112 | 0.910606 |
| NEO-PI-3-A | -0.087 | 0.033 | -0.025 | 0.009 | -2.612 | 0.009184 |
| NEO-PI-3-C | -0.061 | 0.036 | -0.014 | 0.008 | -1.706 | 0.088450 |
|  |  |  |  |  |  |  |
| TEMPS-Hyperthymic Temperament  R=0.59 R²= 0.35 Adjusted R²= 0.35 F(5.728)=79.853 p<0.0000  Std.Error of estimate: 3.6198 | | | | | | |
| Intercept |  |  | 5.015 | 1.981 | 2.532 | 0.011562 |
| NEO-PI-3-E | 0.515 | 0.036 | 0.145 | 0.010 | 14.309 | 0.000000 |
| NEO-PI-3-A | -0.207 | 0.033 | -0.059 | 0.009 | -6.357 | 0.000000 |
| NEO-PI-3-N | -0.151 | 0.035 | -0.035 | 0.008 | -4.359 | 0.000015 |
| NEO-PI-3-C | 0.092 | 0.035 | 0.022 | 0.008 | 2.625 | 0.008853 |
| NEO-PI-3-O | -0.065 | 0.032 | -0.017 | 0.009 | -2.005 | 0.045346 |
|  |  |  |  |  |  |  |
| TEMPS-IrriAdditional Table Temperament  R= 0.63 R²= 0.40 Adjusted R²= 0.40 F(4.729)=123.53 p<0.0000  Std.Error of estimate: 2.9570 | | | | | | |
| Intercept |  |  | 4.759 | 1.546 | 3.078 | 0.002159 |
| NEO-PI-3-N | 0.470 | 0.033 | 0.092 | 0.006 | 14.178 | 0.000000 |
| NEO-PI-3-A | -0.389 | 0.031 | -0.094 | 0.008 | -12.455 | 0.000000 |
| NEO-PI-3-C | 0.057 | 0.034 | 0.011 | 0.007 | 1.704 | 0.088766 |
| NEO-PI-3-E | 0.050 | 0.032 | 0.012 | 0.008 | 1.581 | 0.114250 |
|  |  |  |  |  |  |  |
| TEMPS-Anxious Temperament  R= 0.66 R²= 0.44 Adjusted R²= 0.43 F(4.729)=141.58 p<0.0000  Std.Error of estimate: 4.1495 | | | | | | |
| Intercept |  |  | -9.384 | 2.054 | -4.568 | 0.000006 |
| NEO-PI-3-N | 0.666 | 0.032 | 0.188 | 0.009 | 20.733 | 0.000000 |
| NEO-PI-3-C | 0.124 | 0.031 | 0.036 | 0.009 | 3.997 | 0.000071 |
| NEO-PI-3-O | -0.071 | 0.030 | -0.023 | 0.010 | -2.347 | 0.019181 |
| NEO-PI-3-E | -0.060 | 0.034 | -0.021 | 0.012 | -1.780 | 0.075473 |
|  |  |  |  |  |  |  |
| TEMPS-Depressive Temperament  R= 0.68 R²= 0.46 Adjusted R²= 0.46 F(7.726)=89.847 p<0.0000  Std.Error of estimate: 2.4554 | | | | | | |
| Intercept |  |  | 7.949 | 1.097 | 7.244 | 0.000000 |
| TCI-HA | 0.354 | 0.036 | 0.194 | 0.020 | 9.763 | 0.000000 |
| TCI-SD | -0.321 | 0.039 | -0.159 | 0.019 | -8.172 | 0.000000 |
| TCI-NS | -0.149 | 0.036 | -0.087 | 0.021 | -4.095 | 0.000047 |
| TCI-ST | 0.120 | 0.030 | 0.068 | 0.017 | 4.059 | 0.000055 |
| TCI-PS | 0.114 | 0.029 | 0.205 | 0.053 | 3.896 | 0.000107 |
| TCI-CO | 0.140 | 0.033 | 0.085 | 0.020 | 4.284 | 0.000021 |
| TCI-RD | -0.129 | 0.036 | -0.112 | 0.031 | -3.588 | 0.000356 |
|  |  |  |  |  |  |  |
| TEMPS-Cyclothymic Temperament  R= 0.63 R²= 0.39 Adjusted R²= 0.39 F(6.727)=78.589 p<0.0000  Std.Error of estimate: 3.5259 | | | | | | |
| Intercept |  |  | 8.977 | 1.510 | 5.946 | 0.000000 |
| TCI-SD | -0.360 | 0.040 | -0.241 | 0.027 | -9.070 | 0.000000 |
| TCI-ST | 0.184 | 0.031 | 0.141 | 0.024 | 5.906 | 0.000000 |
| TCI-HA | 0.186 | 0.038 | 0.138 | 0.028 | 4.838 | 0.000002 |
| TCI-NS | 0.229 | 0.038 | 0.181 | 0.030 | 6.045 | 0.000000 |
| TCI-RD | -0.189 | 0.036 | -0.221 | 0.042 | -5.269 | 0.000000 |
| TCI-PS | 0.084 | 0.031 | 0.204 | 0.076 | 2.692 | 0.007275 |
|  |  |  |  |  |  |  |
| TEMPS-Hyperthymic Temperament  R= 0.62 R²= 0.39 Adjusted R²= 0.38 F(5.728)=92.874 p<0.0000  Std.Error of estimate: 3.5196 | | | | | | |
| Intercept |  |  | 12.389 | 1.199 | 10.329 | 0.000000 |
| TCI-HA | -0.501 | 0.031 | -0.368 | 0.023 | -16.250 | 0.000000 |
| TCI-ST | 0.230 | 0.030 | 0.175 | 0.023 | 7.623 | 0.000000 |
| TCI-NS | 0.124 | 0.031 | 0.098 | 0.025 | 4.006 | 0.000068 |
| TCI-PS | 0.128 | 0.031 | 0.310 | 0.075 | 4.125 | 0.000041 |
| TCI-CO | -0.046 | 0.030 | -0.037 | 0.024 | -1.526 | 0.127547 |
|  |  |  |  |  |  |  |
| TEMPS-Irritability Temperament  R= 0.61 R²= 0.37 Adjusted R²= 0.37 F(7.726)=62.085 p<0.0000  Std.Error of estimate: 3.0357 | | | | | | |
| Intercept |  |  | 10.615 | 1.357 | 7.825 | 0.000000 |
| TCI-SD | -0.253 | 0.043 | -0.143 | 0.024 | -5.949 | 0.000000 |
| TCI-CO | -0.259 | 0.035 | -0.180 | 0.024 | -7.370 | 0.000000 |
| TCI-NS | 0.249 | 0.039 | 0.167 | 0.026 | 6.333 | 0.000000 |
| TCI-PS | 0.126 | 0.032 | 0.259 | 0.065 | 3.974 | 0.000078 |
| TCI-RD | -0.146 | 0.039 | -0.145 | 0.039 | -3.748 | 0.000192 |
| TCI-HA | 0.143 | 0.039 | 0.089 | 0.025 | 3.648 | 0.000283 |
| TCI-ST | 0.071 | 0.032 | 0.046 | 0.021 | 2.205 | 0.027743 |
|  |  |  |  |  |  |  |
| TEMPS-Anxious Ttemperament  R= 0.70 R²= 0.49 Adjusted R²= 0.49 F(5.728)=141.67 p<0.0000  Std.Error of estimate: 3.9406 | | | | | | |
| Intercept |  |  | 5.955 | 1.483 | 4.016 | 0.000065 |
| TCI-HA | 0.429 | 0.034 | 0.388 | 0.030 | 12.798 | 0.000000 |
| TCI-SD | -0.298 | 0.034 | -0.244 | 0.028 | -8.736 | 0.000000 |
| TCI-PS | 0.161 | 0.027 | 0.480 | 0.082 | 5.872 | 0.000000 |
| TCI-ST | 0.139 | 0.028 | 0.130 | 0.027 | 4.881 | 0.000001 |
| TCI-RD | -0.065 | 0.028 | -0.093 | 0.039 | -2.368 | 0.018128 |
|  |  |  |  |  |  |  |
| NEO-PI-3-N  R= 0.72 R²= 0.52 Adjusted R²= 0.52 F(5.728)=163.15 p<0.0000  Std.Error of estimate: 13.502 | | | | | | |
| Intercept |  |  | 79.591 | 2.126 | 37.430 | 0.000000 |
| TEMPS-Anx | 0.304 | 0.039 | 1.080 | 0.139 | 7.788 | 0.000000 |
| TEMPS-Cycl | 0.201 | 0.038 | 0.873 | 0.165 | 5.284 | 0.000000 |
| TEMPS-Hyper | -0.233 | 0.028 | -1.016 | 0.121 | -8.367 | 0.000000 |
| TEMPS-Irrit | 0.202 | 0.034 | 1.034 | 0.176 | 5.860 | 0.000000 |
| TEMPS-Depr | 0.066 | 0.036 | 0.390 | 0.212 | 1.842 | 0.065910 |
|  |  |  |  |  |  |  |
| NEO-PI-3-E  R= 0.60 R²= 0.36 Adjusted R²= 0.35 F(4.729)=104.02 p<0.0000  Std.Error of estimate: 12.781 | | | | | | |
| Intercept |  |  | 97.972 | 2.012 | 48.686 | 0.000000 |
| TEMPS-Hyper | 0.478 | 0.032 | 1.701 | 0.113 | 15.017 | 0.000000 |
| TEMPS-Depr | -0.215 | 0.038 | -1.029 | 0.184 | -5.610 | 0.000000 |
| TEMPS-Irrit | -0.140 | 0.038 | -0.586 | 0.158 | -3.709 | 0.000224 |
| TEMPS-Cycl | 0.049 | 0.043 | 0.174 | 0.152 | 1.143 | 0.253484 |
|  |  |  |  |  |  |  |
| NEO-PI-3-O  R= 0.21 R²= 0.04 Adjusted R²= 0.04 F(4.729)=8.8458 p<.00000  Std.Error of estimate: 16.525 | | | | | | |
| Intercept |  |  | 104.237 | 2.601 | 40.070 | 0.000000 |
| TEMPS-Depr | -0.117 | 0.051 | -0.593 | 0.258 | -2.299 | 0.021777 |
| TEMPS-Hyper | 0.084 | 0.039 | 0.317 | 0.148 | 2.147 | 0.032160 |
| TEMPS-Cycl | 0.138 | 0.049 | 0.516 | 0.184 | 2.804 | 0.005185 |
| TEMPS-Anx | -0.127 | 0.053 | -0.389 | 0.161 | -2.424 | 0.015580 |
|  |  |  |  |  |  |  |
| NEO-PI-3-A  R= 0.54 R²= 0.29 Adjusted R²= 0.29 F(5.728)=61.861 p<0.0000  Std.Error of estimate: 13.300 | | | | | | |
| Intercept |  |  | 114.820 | 2.095 | 54.817 | 0.000000 |
| TEMPS-Irrit | -0.567 | 0.042 | -2.349 | 0.174 | -13.519 | 0.000000 |
| TEMPS-Depr | 0.300 | 0.044 | 1.420 | 0.209 | 6.806 | 0.000000 |
| TEMPS-Hyper | 0.070 | 0.034 | 0.245 | 0.120 | 2.049 | 0.040819 |
| TEMPS-Cycl | -0.091 | 0.046 | -0.319 | 0.163 | -1.963 | 0.049973 |
| TEMPS-Anx | 0.077 | 0.048 | 0.221 | 0.137 | 1.614 | 0.106911 |
|  |  |  |  |  |  |  |
| NEO-PI-3-C  R= 0.44 R²= 0.19 Adjusted R²= 0.18 F(5.728)=34.996 p<0.0000  Std.Error of estimate: 17.316 | | | | | | |
| Intercept |  |  | 108.626 | 2.727 | 39.832 | 0.000000 |
| TEMPS-Cycl | -0.344 | 0.050 | -1.468 | 0.212 | -6.930 | 0.000000 |
| TEMPS-Hyper | 0.318 | 0.036 | 1.363 | 0.156 | 8.751 | 0.000000 |
| TEMPS-Depr | 0.226 | 0.047 | 1.304 | 0.272 | 4.801 | 0.000002 |
| TEMPS-Irrit | -0.185 | 0.045 | -0.932 | 0.226 | -4.119 | 0.000042 |
| TEMPS-Anx | 0.086 | 0.051 | 0.299 | 0.178 | 1.678 | 0.093713 |
|  |  |  |  |  |  |  |
| NEO-PI-3-N  R= 0.73 R²= 0.54 Adjusted R²= 0.53 F(6.727)=144.38 p<0.0000  Std.Error of estimate: 13.290 | | | | | | |
| Intercept |  |  | 80.495 | 5.690 | 14.146 | 0.000000 |
| TCI-HA | 0.494 | 0.033 | 1.587 | 0.107 | 14.809 | 0.000000 |
| TCI-SD | -0.297 | 0.034 | -0.862 | 0.100 | -8.613 | 0.000000 |
| TCI-NS | 0.187 | 0.033 | 0.643 | 0.113 | 5.690 | 0.000000 |
| TCI-ST | 0.075 | 0.027 | 0.248 | 0.090 | 2.752 | 0.006078 |
| TCI-RD | -0.101 | 0.031 | -0.512 | 0.158 | -3.239 | 0.001253 |
| TCI-PS | 0.062 | 0.027 | 0.654 | 0.285 | 2.293 | 0.022111 |
|  |  |  |  |  |  |  |
| NEO-PI-3-E  R= 0.63 R²= 0.40 Adjusted R²= 0.40 F(6.727)=82.728 p<0.0000  Std.Error of estimate: 12.366 | | | | | | |
| Intercept |  |  | 89.754 | 4.214 | 21.298 | 0.000000 |
| TCI-HA | -0.386 | 0.031 | -1.009 | 0.080 | -12.634 | 0.000000 |
| TCI-RD | 0.304 | 0.038 | 1.259 | 0.156 | 8.057 | 0.000000 |
| TCI-ST | 0.076 | 0.030 | 0.205 | 0.081 | 2.538 | 0.011361 |
| TCI-PS | 0.083 | 0.031 | 0.714 | 0.265 | 2.689 | 0.007338 |
| TCI-NS | 0.103 | 0.037 | 0.289 | 0.104 | 2.776 | 0.005650 |
| TCI-CO | 0.045 | 0.033 | 0.130 | 0.094 | 1.378 | 0.168585 |
|  |  |  |  |  |  |  |
| NEO-PI-3-O  R= 0.47 R²= 0.22 Adjusted R²= 0.21 F(6.727)=34.830 p<0.0000  Std.Error of estimate: 14.934 | | | | | | |
| Intercept |  |  | 54.451 | 4.549 | 11.970 | 0.000000 |
| TCI-NS | 0.334 | 0.042 | 0.991 | 0.125 | 7.951 | 0.000000 |
| TCI-CO | 0.108 | 0.039 | 0.332 | 0.120 | 2.768 | 0.005779 |
| TCI-ST | 0.128 | 0.035 | 0.365 | 0.101 | 3.624 | 0.000311 |
| TCI-RD | 0.132 | 0.043 | 0.577 | 0.190 | 3.040 | 0.002452 |
| TCI-PS | 0.078 | 0.035 | 0.710 | 0.319 | 2.223 | 0.026525 |
| TCI-SD | 0.044 | 0.038 | 0.110 | 0.095 | 1.157 | 0.247513 |
|  |  |  |  |  |  |  |
| NEO-PI-3-A  R= 0.61 R²= 0.38 Adjusted R²= 0.37 F(4.729)=112.76 p<0.0000  Std.Error of estimate: 12.470 | | | | | | |
| Intercept |  |  | 69.919 | 3.916 | 17.854 | 0.000000 |
| TCI-CO | 0.554 | 0.033 | 1.592 | 0.095 | 16.756 | 0.000000 |
| TCI-NS | -0.223 | 0.036 | -0.619 | 0.101 | -6.154 | 0.000000 |
| TCI-HA | 0.072 | 0.031 | 0.186 | 0.080 | 2.332 | 0.019983 |
| TCI-RD | 0.075 | 0.038 | 0.307 | 0.157 | 1.963 | 0.050038 |
|  |  |  |  |  |  |  |
| NEO-PI-3-C  R= 0.65 R²= 0.43 Adjusted R²= 0.42 F(6.727)=92.392 p<0.0000  Std.Error of estimate: 14.537 | | | | | | |
| Intercept |  |  | 94.247 | 6.224 | 15.143 | 0.000000 |
| TCI-SD | 0.332 | 0.038 | 0.946 | 0.109 | 8.642 | 0.000000 |
| TCI-PS | 0.345 | 0.030 | 3.582 | 0.312 | 11.476 | 0.000000 |
| TCI-NS | -0.315 | 0.037 | -1.062 | 0.124 | -8.602 | 0.000000 |
| TCI-RD | 0.096 | 0.035 | 0.480 | 0.173 | 2.777 | 0.005621 |
| TCI-HA | -0.059 | 0.037 | -0.187 | 0.117 | -1.598 | 0.110541 |
| TCI-ST | -0.038 | 0.030 | -0.125 | 0.098 | -1.275 | 0.202872 |
|  |  |  |  |  |  |  |
| TCI-NS  R= 0.47 R²= 0.22 Adjusted R²= 0.21 F(5.728)=41.991 p<0.0000  Std.Error of estimate: 5.0323 | | | | | | |
| Intercept |  |  | 19.995 | 0.793 | 25.229 | 0.000000 |
| TEMPS-Depr | -0.436 | 0.046 | -0.744 | 0.079 | -9.420 | 0.000000 |
| TEMPS-Cycl | 0.333 | 0.049 | 0.421 | 0.062 | 6.833 | 0.000000 |
| TEMPS-Irrit | 0.179 | 0.044 | 0.267 | 0.066 | 4.055 | 0.000056 |
| TEMPS-Anx | -0.113 | 0.050 | -0.117 | 0.052 | -2.265 | 0.023823 |
| TEMPS-Hyper | 0.074 | 0.036 | 0.094 | 0.045 | 2.067 | 0.039091 |
|  |  |  |  |  |  |  |
| TCI-HA  R= 0.73 R²= 0.54 Adjusted R²= 0.53 F(4.729)=214.03 p<0.0000  Std.Error of estimate: 4.1511 | | | | | | |
| Intercept |  |  | 15.603 | 0.652 | 23.934 | 0.000000 |
| TEMPS-Anx | 0.345 | 0.037 | 0.382 | 0.041 | 9.213 | 0.000000 |
| TEMPS-Hyper | -0.391 | 0.027 | -0.532 | 0.037 | -14.448 | 0.000000 |
| TEMPS-Depr | 0.189 | 0.034 | 0.345 | 0.062 | 5.606 | 0.000000 |
| TEMPS-Irrit | 0.054 | 0.031 | 0.086 | 0.049 | 1.736 | 0.082937 |
|  |  |  |  |  |  |  |
| TCI-RD  R= 0.34 R²= 0.12 Adjusted R²= 0.11 F(3.730)=33.952 p<0.0000  Std.Error of estimate: 3.6202 | | | | | | |
| Intercept |  |  | 15.787 | 0.569 | 27.768 | 0.000000 |
| TEMPS-Depr | -0.244 | 0.040 | -0.282 | 0.046 | -6.159 | 0.000000 |
| TEMPS-Hyper | 0.114 | 0.037 | 0.098 | 0.032 | 3.086 | 0.002103 |
| TEMPS-Irrit | -0.110 | 0.037 | -0.111 | 0.038 | -2.926 | 0.003541 |
|  |  |  |  |  |  |  |
| TCI-PS  R= 0.32 R²= 0.10 Adjusted R²= 0.10 F(5.728)=17.655 p<.00000  Std.Error of estimate: 1.7546 | | | | | | |
| Intercept |  |  | 2.202 | 0.276 | 7.968 | 0.000000 |
| TEMPS-Hyper | 0.314 | 0.038 | 0.129 | 0.016 | 8.204 | 0.000000 |
| TEMPS-Depr | 0.208 | 0.050 | 0.116 | 0.028 | 4.200 | 0.000030 |
| TEMPS-Cycl | -0.162 | 0.052 | -0.066 | 0.021 | -3.097 | 0.002029 |
| TEMPS-Anx | 0.206 | 0.054 | 0.069 | 0.018 | 3.841 | 0.000133 |
| TEMPS-Irrit | -0.060 | 0.047 | -0.029 | 0.023 | -1.272 | 0.203638 |
|  |  |  |  |  |  |  |
| TCI-SD  R= 0.66 R²= 0.44 Adjusted R²= 0.43 F(5.728)=114.67 p<0.0000  Std.Error of estimate: 5.0636 | | | | | | |
| Intercept |  |  | 36.491 | 0.797 | 45.760 | 0.000000 |
| TEMPS-Anx | -0.208 | 0.043 | -0.255 | 0.052 | -4.893 | 0.000001 |
| TEMPS-Cycl | -0.239 | 0.041 | -0.357 | 0.062 | -5.768 | 0.000000 |
| TEMPS-Hyper | 0.130 | 0.030 | 0.195 | 0.046 | 4.284 | 0.000021 |
| TEMPS-Irrit | -0.200 | 0.037 | -0.352 | 0.066 | -5.326 | 0.000000 |
| TEMPS-Depr | -0.113 | 0.039 | -0.229 | 0.079 | -2.877 | 0.004127 |
|  |  |  |  |  |  |  |
| TCI-CO  R= 0.47 R²= 0.22 Adjusted R²= 0.21 F(4.729)=51.882 p<0.0000  Std.Error of estimate: 4.8729 | | | | | | |
| Intercept |  |  | 32.139 | 0.767 | 41.892 | 0.000000 |
| TEMPS-Irrit | -0.468 | 0.042 | -0.675 | 0.060 | -11.198 | 0.000000 |
| TEMPS-Hyper | 0.123 | 0.035 | 0.151 | 0.043 | 3.496 | 0.000501 |
| TEMPS-Depr | 0.148 | 0.042 | 0.244 | 0.070 | 3.482 | 0.000527 |
| TEMPS-Cycl | -0.056 | 0.047 | -0.069 | 0.058 | -1.190 | 0.234475 |
|  |  |  |  |  |  |  |
| TCI-ST  R= 0.46 R²= 0.21 Adjusted R²= 0.20 F(5.728)=39.305 p<0.0000  Std.Error of estimate: 5.2498 | | | | | | |
| Intercept |  |  | 5.688 | 0.827 | 6.880 | 0.000000 |
| TEMPS-Hyper | 0.390 | 0.036 | 0.512 | 0.047 | 10.838 | 0.000000 |
| TEMPS-Anx | 0.170 | 0.050 | 0.182 | 0.054 | 3.372 | 0.000787 |
| TEMPS-Depr | 0.134 | 0.047 | 0.236 | 0.082 | 2.868 | 0.004245 |
| TEMPS-Cycl | 0.188 | 0.049 | 0.246 | 0.064 | 3.835 | 0.000136 |
| TEMPS-Irrit | -0.107 | 0.044 | -0.165 | 0.069 | -2.401 | 0.016612 |
|  |  |  |  |  |  |  |
| TCI-NS  R= 0.64 R²= 0.41 Adjusted R²= 0.41 F(4.729)=130.29 p<0.0000  Std.Error of estimate: 4.3589 | | | | | | |
| Intercept |  |  | 16.063 | 1.736 | 9.253 | 0.000000 |
| NEO-PI-3-O | 0.237 | 0.031 | 0.080 | 0.010 | 7.693 | 0.000000 |
| NEO-PI-3-C | -0.449 | 0.032 | -0.133 | 0.010 | -13.968 | 0.000000 |
| NEO-PI-3-E | 0.407 | 0.033 | 0.145 | 0.012 | 12.518 | 0.000000 |
| NEO-PI-3-A | -0.121 | 0.031 | -0.043 | 0.011 | -3.905 | 0.000103 |
|  |  |  |  |  |  |  |
| TCI-HA  R= 0.71 R²= 0.51 Adjusted R²= 0.51 F(3.730)=258.17 p<0.0000  Std.Error of estimate: 4.2608 | | | | | | |
| Intercept |  |  | 4.977 | 2.082 | 2.390 | 0.017087 |
| NEO-PI-3-N | 0.558 | 0.029 | 0.174 | 0.009 | 19.409 | 0.000000 |
| NEO-PI-3-E | -0.313 | 0.028 | -0.120 | 0.011 | -11.071 | 0.000000 |
| NEO-PI-3-A | 0.168 | 0.027 | 0.065 | 0.010 | 6.307 | 0.000000 |
|  |  |  |  |  |  |  |
| TCI-RD  R= 0.54 R²= 0.29 Adjusted R²= 0.29F(5.728)=61.597 p<0.0000  Std.Error of estimate: 3.2440 | | | | | | |
| Intercept |  |  | 0.899 | 1.775 | 0.506 | 0.612714 |
| NEO-PI-3-E | 0.416 | 0.038 | 0.100 | 0.009 | 11.071 | 0.000000 |
| NEO-PI-3-O | 0.204 | 0.034 | 0.047 | 0.008 | 6.031 | 0.000000 |
| NEO-PI-3-C | -0.201 | 0.037 | -0.040 | 0.007 | -5.474 | 0.000000 |
| NEO-PI-3-A | 0.130 | 0.034 | 0.032 | 0.008 | 3.830 | 0.000139 |
| NEO-PI-3-N | -0.065 | 0.036 | -0.013 | 0.007 | -1.808 | 0.070989 |
|  |  |  |  |  |  |  |
| TCI-PS  R= 0.47 R²= 0.22 Adjusted R²= 0.22 F(4.729)=53.095 p<0.0000  Std.Error of estimate: 1.6338 | | | | | | |
| Intercept |  |  | -2.511 | 0.831 | -3.021 | 0.002607 |
| NEO-PI-3-C | 0.541 | 0.038 | 0.052 | 0.004 | 14.322 | 0.000000 |
| NEO-PI-3-N | 0.167 | 0.036 | 0.016 | 0.003 | 4.640 | 0.000004 |
| NEO-PI-3-A | -0.099 | 0.036 | -0.012 | 0.004 | -2.772 | 0.005713 |
| NEO-PI-3-O | 0.048 | 0.033 | 0.005 | 0.004 | 1.474 | 0.140918 |
|  |  |  |  |  |  |  |
| TCI-SD  R= 0.66 R²= 0.44 Adjusted R²= 0.44 F(3.730)=197.56 p<0.0000  Std.Error of estimate: 5.0226 | | | | | | |
| Intercept |  |  | 36.360 | 2.193 | 16.583 | 0.000000 |
| NEO-PI-3-N | -0.533 | 0.030 | -0.184 | 0.010 | -17.605 | 0.000000 |
| NEO-PI-3-C | 0.226 | 0.032 | 0.079 | 0.011 | 7.091 | 0.000000 |
| NEO-PI-3-A | 0.031 | 0.030 | 0.013 | 0.013 | 1.026 | 0.305178 |
|  |  |  |  |  |  |  |
| TCI-CO  R= 0.62 R²= 0.39 Adjusted R²= 0.38 F(5.728)=93.761 p<0.0000  Std.Error of estimate: 4.3106 | | | | | | |
| Intercept |  |  | 7.228 | 2.359 | 3.064 | 0.002266 |
| NEO-PI-3-A | 0.563 | 0.032 | 0.196 | 0.011 | 17.786 | 0.000000 |
| NEO-PI-3-O | 0.165 | 0.032 | 0.054 | 0.010 | 5.240 | 0.000000 |
| NEO-PI-3-N | -0.141 | 0.034 | -0.040 | 0.009 | -4.201 | 0.000030 |
| NEO-PI-3-C | -0.042 | 0.034 | -0.012 | 0.010 | -1.243 | 0.214375 |
| NEO-PI-3-E | 0.037 | 0.035 | 0.013 | 0.012 | 1.048 | 0.295047 |
|  |  |  |  |  |  |  |
| TCI-ST  R= 0.27 R²= 0.07 Adjusted R²= 0.07 F(3.730)=20.332 p<.00000  Std.Error of estimate: 5.6756 | | | | | | |
| Intercept |  |  | -2.027 | 2.307 | -0.879 | 0.379841 |
| NEO-PI-3-O | 0.128 | 0.038 | 0.045 | 0.013 | 3.315 | 0.000961 |
| NEO-PI-3-N | 0.221 | 0.039 | 0.066 | 0.012 | 5.651 | 0.000000 |
| NEO-PI-3-E | 0.175 | 0.042 | 0.065 | 0.015 | 4.172 | 0.000034 |
